# Retinoic acid-loaded PLGA nanocarriers targeting cell cholesterol potentialize the antitumour effect of PD-L1 antibody by preventing epithelial-mesenchymal transition mediated by M2-TAM in colorectal cancer

**DOI:** 10.1016/j.tranon.2023.101647

**Published:** 2023-02-27

**Authors:** Raimundo Fernandes de Araújo Júnior, George A Lira, Timo Schomann, Rômulo S Cavalcante, Natalia Feitosa Vilar, Regina Célia Monteiro de Paula, Raelle Ferreira Gomes, Chih Kit Chung, Carla Jorquera-Cordero, Olena Vepris, Alan B Chan, Luis J. Cruz

**Affiliations:** a Cancer and Inflammation Research Laboratory, Department of Morphology, Federal University of Rio Grande do Norte Natal, RN 59072-970, Brazil; b Post-Graduation Programme in Structural and Functional Biology, Federal University of Rio Grande do Norte, Natal, RN 59072-970, Brazil; c Post-Graduation Programme in Health Science, Federal University of Rio Grande do Norte, Natal, RN 59072-970, Brazil; d Percuros B.V., Leiden, CL 2333, the Netherlands; e Translational Nanobiomaterials and Imaging, Department of Radiology, Leiden University Medical Center, Leiden, ZA 2333, the Netherlands; f Department of Orthopedics, University Medical Center Utrecht, Heidelberglaan 100, Utrecht, CX 3584, the Netherlands; g League Against Cancer from Rio Grande do Norte, Advanced Oncology Center, Natal 59075-740, Brazil; h Post-Graduation Programme in Chemistry, Federal University of Ceará, Fortaleza, CE 60440-900, Brazil; i JeNaCell GmbH, Winzerlaer Straße 2, Jena 07745, Germany

**Keywords:** Immunomodulation, M2-like macrophages, PLGA nanoparticles, Anti-PD-L1, STAT3/NF-κB, Metastasis, Tumour microenvironment

## Abstract

Tumour-associated macrophages (TAMs) often promote cancer progression through immunosuppression in the tumour microenvironment (TME). However, the signalling pathways crosstalk responsible for this mechanism remain unclear. The aim of our study was to investigate whether the interaction between TAMs and colorectal cancer cells could be down-regulated by nanoparticles (NPs) loaded with retinoic acid (RA) and coated with cholesterol (CHO), in combination with an anti-PD-L1 immune checkpoint inhibitor. Tumours were evaluated by qRT-PCR and immunohistochemistry from allographic tumour growth model. In addition, human tumours were evaluated by Tissue Microarray (TMA) and immunohistochemistry. Complementary analysis of epithelial-mesenchymal transition, cell migration, and macrophage polarisation were evaluated in vitro. We showed that the IL-10R/IL-10 axis is involved in overstimulation of the STAT3 pathway as well as downregulation of the NF-κB signalling pathway, which supports a loop of immunosuppressive cytokines that induces the M2-TAM phenotype. Furthermore, our combined findings suggest that the upregulation of STAT3/NF-κB pathways crosstalk mediated by immunosuppressive cytokines, such as IL-10/PD-L1/TGF-β, via M2-TAMs in the TME, leads to immunosuppression and epithelial-mesenchymal-transition of the colorectal cancer for stimulating Vimentin, CXCL12 and CD163 in the primary tumours. Importantly, NPs holding RA and coated with CHO in combination with anti-PD-L1 were more efficient in blocking this signalling pathway. These results contribute to our understanding of the immunological mechanisms, especially the re-educating of TAMs, and provide a novel management strategy for aggressive colorectal cancers using anti-PD-L1-conjugated nanocarriers.

## Highlights

• Understanding the molecular mechanisms of TAM re-education in the tumor microenvironment.
• NPs-mediated immunosuppression disruption between immune and cancer reduces metastasis.
• Combine new immunotherapeutic strategies to fight colorectal cancer.

## Graphical abstract

**Figure:**
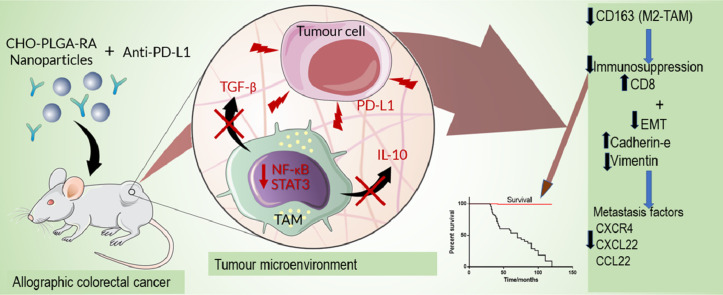

## Introduction

Inflammation-induced cancers represent a broad spectrum of diseases schematically related to two stimulatory factors: an intrinsic pathway, which causes inflammation and tumorigenesis by genetic alterations; and an extrinsic pathway driven by immune cells, such as macrophages, in a chronic inflammatory process, which in-creases the risk of cancer [1].

Worldwide, colorectal cancer (CRC) is the third most common cancer, in which incident cases and deaths were higher among males than females at the age of 80-84 years. The highest rates being observed in the oldest age group (≥95 years) for both sexes [2]. Both environmental and genetic factors influence the development of CRC, especially in producing a prominent inflammatory response, with >70% of cases classified as sporadic [3].

Macrophages may be important targets for nanomedicine because they are the main mediators of intestinal immune homeostasis and play an important role in inflammation-associated CRC by modulating intrinsic factors and the extrinsic aspects of the tumour, which makes it resistant to treatment [4]. In addition, macrophages have high cellular plasticity and are capable of dedifferentiation according to the inflammation level in the tumour microenvironment (TME) [5]. Macrophages can be differentiated into two subsets: (i) pro-inflammatory (classically activated M1 macrophages via interferon-γ); and (ii) anti-inflammatory (alternatively activated M2 macrophages via interleukin (IL)-4 and IL-13) [6]. However, in cancer, macrophages assume other functions: M1 macrophages exert tumoricidal effects, whereas M2 macrophages or tumour-associated macrophages (TAMs) promote tumour progression by stimulating angiogenesis, migration and invasion of tumour cells, and suppression of anti-tumour immune responses [6,7].

In recent years, therapies aimed at the downregulation of immunosuppression within the TME or reducing tumour escape by employing immune checkpoint block-ers. The expression of immune checkpoints, such as programmed cell death-1 ligand 1 (PD-L1), in tumour cells and tumour-infiltrating immune cells (ICs) is highly associated with M2-TAMs, which play important roles in regulating the anti-tumour T cell response [8]. Immune cells, such as M2-TAMs, often paradoxically promote cancer progression by enabling tumour cells to escape from immunosurveillance or by establishing immunosuppression in the TME through a process known as “cancer immunoediting” [9]. Moreover, M2-TAMs play a crucial role in tumour progression due to their influence on epithelial-mesenchymal transition (EMT) of the primary tumour by means of in-inflammatory cytokines and growth factors, such as IL-6, IL-10, and TGFβ [10], [11], [12]. M2-TAMs are influenced by malignant tumour cells via the regulation of nuclear factor kappa B (NF-κB) and often conditioned to change their cancer-fighting phenotype towards a cooperative, tumour-promoting profile via immunosuppressive cytokines, thereby, stimulating metastasis [13,14]. In addition, signal transducer and activator of transcription 3 (STAT3) is constitutively activated in a wide variety of solid tumours including breast cancer, prostate cancer, CRC, melanoma, and brain cancer [15,16]. The interaction of IL-10 with IL-10R triggers the Jak-STAT signalling pathway, leading to STAT1 and STAT3 activation and to NF-κB inhibition. STAT3 and NF-kB are critical for IL-10 effects on macrophage polarization [11]. STAT3 pathway supports the EMT process by stimulating specific transcription factors [17] In previous studies, we have shown that the NF-κB/STAT3 crosstalk mediated immunosuppression by M2-TAMs in the TME of breast cancer [18].

In this study, we investigated whether STAT3/NF-κB-mediated EMT and immunosuppression are orchestrated by M2-TAM via a loop of immunosuppressive cytokines in the TME of the CRC. Importantly, we suggested a drug delivery system based on poly(lactic-co-glycolic acid) (PLGA) nanoparticles (NPs) loaded with retinoic acid (RA) and coated with cholesterol (CHO), in combination with the immune checkpoint inhibitor anti-PD-L1, which is a powerful blockage of EMT and M2-TAM-induced immunosuppression. RA [19,20] reduces inflammation and cytokine production in clinical treatments by supressing signalling pathways involved in the production of inflammatory cytokines, such as the NF-κB pathway, which is critical for expressing various inflammatory genes [[21], [22], [23]]. Although its role in educating TAMs remain unclear, our investigation of the STAT3/NF-κB pathway crosstalk elucidated several mechanisms of this process, which resulted in important findings in vivo when RA-loaded NPs were combined with the immune checkpoint blocker anti-PD-L1. Due to the high level of cell metabolism in cancer, the loss of cholesterol-rich membrane micro-domains by upregulation of the cholesterol efflux transporters in macrophages causes an amplification of IL-4 receptor activity which leads to M2-TAM phenotype in TME [24,25] . On the other hand, the high rate of proliferation and the need for nutrients for the formation of new cell membranes lead to the overexpression of cholesterol receptors like SR-B1 or LDL on surface of tumor cells [26,27] . Therefore, CHO is an important target of our drug delivery system. In view of the scarce alternatives to treat aggressive tumours in more advanced stages of cancer, new protocols need to be developed beyond the state of the art to target M2-TAMs in the TME.

## Material and methods

### Reagents and antibodies

Thermo Fisher Scientific supplied mouse anti-E-cadherin, mouse anti-vimentin, mouse anti-catenin, mouse anti-galectin, mouse anti-PD-L1, mouse anti-TGF, mouse anti-NF-κB antibodies, mouse anti-STAT3, and Hoechst (Waltham, MA, USA). eBio-science provided mouse anti-CD163-PerCP, mouse anti-CD206-PerCP, mouse anti-Ki-67-APC, mouse anti-CD68-FITC, and mouse anti-IL-10-FITC (San Diego, CA, USA). BD Cytofix/Cytoperm Fixation/Permeabilization Solution Kit with GolgiPlug™(No. BDB555028, San Diego, CA, USA). PeproTech delivered recombinant mouse IL-4, IFN-, and anti-IL-10 receptor (abIL-10R) inhibitors (Rocky Hill, NJ, USA).

Mouse anti-STAT3 (Invitrogen, cat number MA1-13042), rabbit anti-NF-κB (Life technologies, cat number 510500), mouse anti-PD-L1/CD274 (Proteintech, cat number 66248-1-Ig), and mouse anti-TGF (Invitrogen, cat number MA5-15065) were used for immunoblotting, and so were secondary antibodies goat anti-mouse and goat anti-rabbit Alexa® Fluor 555 (Thermo Fisher Scientific, Waltham). For immunoblotting, rabbit anti-actin (LI-COR Biosciences, Lincoln, Nebraska, USA) was used. The anti-mouse PD-L1 checkpoint blocker was purchased from InVivoPlus (B7-H1, Bio cell, USA).

### Preparation of PLGA nanoparticles

First, following the solvent evaporation-extraction method [25], the PLGA NPs were synthesized in an oil/water emulsion [25]. The emulsification solvent extraction/evaporation technique, we anticipated, would enhance the encapsulation yield of hydrophobic (RA) molecules at the same time. 100 mg PLGA (Corbion, Amsterdam, The Netherlands) were dissolved in 3 mL dichloromethane, together with 20 mg RA and 0.5 mg IR-780 iodide (Sigma-Aldrich, St. Louis, MO, USA). The NPs constituent solution was poured into 25 mL of aqueous 2.5 % (w/v) polyvinyl alcohol (PVA) and emulsified using a sonicator (250 watt, Sonifier 250; Branson, Danbury, CT, USA). PVA worked as a surfactant molecule, stabilizing the emulsion nanoparticles, preventing them from aggregation and coalescing with each other. Additionally, PVA acts as an effective stabilizer, and the PVA surfactant molecules helped us obtain small particle sizes and a narrow size distribution. PVA remained at extremely low quantities on the surface of nanoparticles [25]. The solution was transferred to a new vial containing an air-dried 20 mg CHO (Avati Polar Lipids, Alabaster, AL, USA) solution dissolved in 0.4 mL chloroform and homogenized by sonication. The NPs were centrifuged and lyophilized for 3 days after the solvent was evaporated. Reverse phase high-performance liquid chromatography (RP-HPLC) was used to determine the concentration of each encapsulated ingredient, as described elsewhere [25]. Afterwards, three nanoparticle systems were synthesized holding or not RA and coated or not CHO. They were named such as: PLGA NPs holding RA and coated with CHO (NP3); PLGA NPs holding RA but not coated with CHO (NP4); PLGA NPs without RA but coated with CHO (NP8). The final formulation of our NPs was NP3, NP4 and NP8.

$$\text{Loading}\;\text{retinoic}\;\text{acid}\;\text{content}=\frac{mass\;of\;retinoic\;acid\;in\;nanoparticles\left(g\right)}{mass\;of\;naniparticle\left(mg\right)}$$

$$Loading\;retinoic\;acid\;efficiency\;\left(\%\right)=\frac{mass\;of\;retnoic\;acid\;in\;nanoparticle}{mass\;of\;feeding\;retinoic\;acid}x100$$

### Physicochemical properties of PLGA NPs

PLGA NPs were characterized by average size, polydispersity index, and surface charge (zeta-potential) by dynamic light scattering. PLGA NP samples were measured for size using a Zetasizer (Nano ZS, Malvern Ltd., Worcestershire, UK), and were analysed for surface charge by laser Doppler electrophoresis on the same device. The hydrodynamic diameter and the polydispersity index were analysed as stability parameters of the nanoparticles loaded with retinoic acid (NP3 e NP4) [28]. These two parameters were investigated in water, and the sample were kept refrigerated at 4°C.

### In-vitro drug release

The in vitro drug release of RA from PLGA nanoparticles was evaluated in phosphate-buffered saline (PBS) with pH = 7.4 containing 3%ethanol, as release medium. Briefly, 1 mg of the freeze-dried nanoparticles (NP3)PLGA-RA-CHO and NP4(PLGA-RA) were resuspended in PBS (1 mg/mL) containing 3% of ethanol, triplicate for each formulation, and incubated at 37°C under continuous shaking (300 rpm) as described by Oliveira et al [29]. The nanoparticles solution was introduced into a cellulose membrane ((14.000 g/mol) and dialyzed against 20 mL of (PBS) with pH = 7.4 containing 3%ethanol 37 °C. At predetermined time points 3mL aliquots were removed and 3 mL of fresh, warm PBS added to the release medium. The concentration of RA in the supernatant was measured spectrophotometrically at λ = 349 nm (Shimadzu UV-1800).

### Scanning electron microscopy (SEM)

The morphology Analyses of the NP3 and NP4 nanoparticles were performed in a Scanning Electron Microscope (Quanta 450 – FEG) with EDS/EBSD. Before the analysis, the samples were metallized in a Metallizer equipment (QT150 ES – Quorum)

### Cell culture and cell lines

The American Type Culture Collection provided the murine CRC cell line CT-26 and the mouse macrophage cell line RAW 264.7. (Rockville, MD, USA). Cell lines were grown in Dulbecco's modified Eagle's medium (DMEM) supplemented with 1% antibiotics (penicillin/streptomycin) and 10 % fetal bovine serum (Gibco Laboratories, Grand Island, NY, USA) (FBS). The cells were kept at a temperature of 37°C in a humidified incubator with 5% CO2. Cells were passaged twice a week by removing adhering cells in phosphate-buffered saline with trypsin/EDTA (Gibco Laboratories, Grand Island, NY, USA) (PBS).

### Cell viability assays

CT-26 and RAW 264.7 cells were cultured (3 × 10^3^ cells/well) in a 96-well plate. After 24 hours, RA, CHO, NP3-(RA)-CHO, NP4-(RA), NP8-CHO, and anti-IL-10R inhibitor were added at three different concentrations as selected in a pilot study, such as: (i.e., RA: 0.5 µM, 1 µM, and 5 µM; CHO: 1 µg/ml, 5 µg/ml, and 10 µg/ml; NP3: 1 µg/ml, 2.5 µg/ml, and 5 µg/ml; NP4: 1 µg/ml, 2.5 µg/ml, and 5 µg/ml; NP8: 1 µg/ml, 2.5 µg/ml, and 5 µg/ml; abIL-10R: 0.6 µg/ml, 1.2 µg/ml, and 2.4 µg/ml). All compounds were maintained in the dark at -20°C and dissolved in PBS. After another 24 hours and 48 hours, 20 μl/well of CellTiter 96 Aqueous One Solution (MTS) solution (Promega, Madison, WI, USA) was added and incubated with 5 % CO2 at 37°C for 3 hours. All experiments were carried out in triplicate, with the average of the duplicates shown. 25 % DMSO was utilized as a positive control (“death”), and culture media without medication was employed as a negative control (“live”). Absorbance was measured at 490 nm.

### Internalization of nanoparticles by cells and fluorescence imaging

The RAW 264.7 and CT-26 cells were plated and treated with NP3-(RA)-CHO e NP4-(RA) at 10 μg/mL. After 4 e 24h, the cells were stained with DiD and DAPI. For the visualization of nanoparticles (green) and nucleus (blue), a Leica DM5500 B fluorescence microscope equipped with a Leica DFC365 FX digital camera (Leica) was used. Digital images were acquired, analyzed, and stored using Leica Application Suite X (LAS X) software.

### RAW 264.7 cell polarization towards M1 and M2 macrophages

RAW 264.7 cells (5 × 10^5^ cells/well in a 12-well plate) were grown in complete medium containing 10 % FBS, supplemented with 20 ng/ml IL-4 for 24 hours to induce M2 TAM polarization. RAW 264.7 cells were washed three times with serum-free medium after 24 hours and cultivated in serum-free medium supplemented with IL-4 for 48 hours [11]. RAW 264.7 cells were harvested and used as M2-polarized TAMs after 48 hours of serum deprivation. RAW 264.7 supernatant was collected throughout the polarization process and used as M2-polarized TAM-conditioned media (CM) [11]. RAW 264.7 cells (5 × 10^5^ cells/well in a 12-well plate) were grown in medium containing 10 % FBS, supplemented with 1 ng/ml LPS and 0.1 ng/ml IFN-γ for 24 hours to induce polarization to M1-TAMs. After 24 hours, RAW 264.7 cells were washed three times with serum-free medium and cultured in serum-free medium for another 48 hours. To assess the effect of compounds (NP3 (RA)-CHO; NP4-(RA); NP8-CHO, and abIL-10R) on the polarization state of M2 macrophages, RAW 264.7 cells were first cultured in the presence of IL-4, 10% serum, and all compounds, as described above for 24 hours. After 24 hours, the cells were washed three times with serum-free medium, resuspended in serum-free medium, and cultured for another 48 hours. Cells morphology, as an indication of M2 polarisation, was analysed using a Telaval 31 light microscope (ZEISS, Oberkochen, Germany).

### Flow cytometry

After incubation and treatment with 1 µM RA, 5μg/ml CHO, 2.5 μg/ml NP3 (RA)-CHO, 2.5 μg/ml NP4-RA, 2.5 μg/ml NP8-CHO, 1.2 μg/ml abIL-10R, and 2.5 μg/ml NP3 (RA)-CHO+1.2 μg/ml abIL-10R for 48 hours, RAW 264.7 cells (1.5 × 104 cells/well in a 12-well plate) were collected with a cell scraper and blocked with 0.5 % BSA in PBS for 45 minutes. To evaluate whether the cells show characteristics of M1 or M2 macrophages, RAW 264.7 cells were labelled with anti-mouse CD68-FITC (1:1000) or CD163-PerCP antibody (1:1000), respectively [11]. Next, staining of intracellular IL-10 was performed. For this purpose, cells were fixed in 2% paraformaldehyde (PFA, BD Cytofix/Cytoperm Fixation/Permeabilization Solution Kit with GolgiPlug™) for 15 minutes at room temperature. Next, cells were washed and permeabilized with 0.1% Triton X-100 for 5 minutes and, subsequently, blocked by 0.5% BSA in PBS for 45 minutes. For intracellular staining, permeabilized cells were incubated using a cytofix/cytoperm kit (BD Biosciences) for 1 h. The anti-mouse IL-10-FITC antibody (1:1000) was applied for 60 minutes at 4°C for intracellular IL-10 staining. After a final washing step, the cells were analysed by flow cytometry [11].

### Enzyme-linked Immunosorbent assay (ELISA)

Cells were incubated and treated with 2.5 μg/ml NP3 (RA)-CHO, 1.2 μg/ml abIL-10R, and 2.5 μg/ml NP3 (RA)-CHO+1.2 μg/ml abIL-10R for 48 hours. After 48 hours of incubation, the supernatants were collected and murine IL-10 and IL-12 levels were determined by means of a LEGEND MAX™ Mouse IL-10/IL-12 ELISA Kit (Bio-Legend, San Diego, CA, USA) according to the protocol of the manufacturer. Experiments were performed in triplicate and repeated twice.

### Indirect Co-culture of CRC cells and M2-polarized TAMs

CT-26 cells (1 × 10^4^ cells/well) were grown in DMEM containing 10 % FBS in 12-well plates (BD Bioscience). In DMEM with 10 % FBS, M2-polarized TAMs (5 × 104 cells/insert) were seeded into the upper chamber of a transwell insert with an 8 μm pore size (Corning Inc., Corning, NY, USA). The next day, the M2-polarized TAMs were treated with 2.5 μg/ml NP3 (RA)-CHO in the presence or absence of 1.2 μg/ml anti-IL-10R inhibitor, and the cells were cultivated for another 48 hours at 37°C in a humidified incubator with 5% CO2. A Telaval 31 light microscope (ZEISS) was used to examine the morphology of the cells. CT-26 cells were collected after treatment and fixed with 2% PFA and permeabilized with 0.1 % Triton X-100. Next, the cells were incubated with mouse anti-Ki-67-APC (1:100) or mouse anti-IL-10-FITC (1:100) at 4°C for 60 minutes and, subsequently, analysed using flow cytometry [11].

### Wound healing assay

The method used for the wound healing assay has been described previously [11,28]. Briefly, the CT-26 cells were seeded in 12-well plates and incubated in DMEM containing 10% FBS until they reached a confluence of 70%. The sub-confluent cell mono-layer was incubated with 10 μg/ml mitomycin C for 2 hours to inhibit cell proliferation. Next, wounds were introduced using a plastic pipette tip. Next, the cells were washed with PBS twice and cultured with M2-TAM-CM and serum-free DMEM (ratio 1:1), together with treatment of either 2.5 μg/ml NP3 (RA)-CHO or 2.5 μg/ml NP3 (RA)-CHO+1.2 μg/ml abIL-10R. After 4 hours and 24 hours, the area of wound induction was monitored using an Evos M5000 (Thermo Fisher Scientific). Using Fiji analytic software (https://fiji.sc/), the wound area was designated and the number of cells that migrated into the area of wound induction was measured at both time points (i.e., 4 hours and 24 hours). In each trial, two wound regions were examined, and the procedure was repeated twice.

### Transwell migration assay

The method used for the transwell migration assay has been described previous-ly [11]. Cell migration was examined with the two-chamber assay using a transwell co-culture system (Corning). Both 1 × 104 CT-26 cells and 5 × 104 RAW 264.7 cells were seeded into the upper chamber of a transwell culture plate (insert) and on the bottom of the 12-well plate, respectively, and incubated with DMEM containing 10% FBS for 24 hours. Next, cells were incubated with 10 μg/ml mitomycin C for 2 hours and washed twice with PBS. Afterwards, CT-26 cells were incubated with serum-free DMEM and treated with 2.5 μg/ml NP3 (RA)-CHO and 2.5 μg/ml NP3 (RA)-CHO+1.2 μg/ml abIL-10R for 48 hours. The cell suspension in the upper chamber was aspirated for nuclear staining. The upper surface of the filter was meticulously cleaned with cotton plugs to remove any cells that did not migrate through the membrane. DAPI was used to label the nuclei of the cells that moved through the membrane (Thermo Fisher Scientific). With an Evos M5000, three representative photos of two technical duplicates were taken. Fiji analysis software (https://fiji.sc/) was used to count the migratory and invasive cells that travelled through the 5-μm-sized pores.

### Immunofluorescence

For evaluation of EMT, CT-26 cells were plated on cover glasses at a cell density of 5 × 104 cells/coverslip in 12-well plates (total volume of 1 ml). After 24 hours, cells were treated with CM/serum-free DMEM (ratio 1:1), 2.5 μg/ml NP3 (RA)-CHO, 1.2 μg/ml abIL-10R, and 2.5 μg/ml NP3 (RA)-CHO+1.2 μg/ml ab-IL-10R for 48 hours. All cells were fixed in PBS with 1 % PFA and kept at 4°C until needed. Fixed specimens were rinsed with 0.05 % Tween-20 (Sigma Aldrich) in PBS for 5 minutes before being permeabilized with 0.1 % Triton X-100 (Sigma-Aldrich) in PBS for 10 minutes. Next, the cells were then rinsed with 0.05 % Tween-20 in PBS for 5 minutes before being incubated for 30 minutes in a blocking solution comprising 0.1 % Triton X-100 and 5% normal goat serum (Dako, Glostrup, Denmark). The specimens were treated in blocking solution with the primary antibodies at 4°C overnight. Anti-vimentin (1:100), anti-E-cadherin (1:100) and STAT3 (1:100) were the primary antibodies employed. After washing, the specimens were incubated in blocking solution for 10 minutes, then incubated in blocking solution (1:300) for 60 minutes with goat anti-mouse or goat anti-rabbit Alexa® Fluor 555-conjugated secondary antibodies. Nuclear staining was done with DAPI (1:1000; Life Technologies, USA) in PBS. Each batch of samples included relevant positive and negative controls. A Leica DM5500 B fluorescence microscope with a Leica DFC365 FX digital camera was used to analyse the specimens. Leica Application Suite X (LAS X) software was used to capture and store digital photographs. Each batch of samples included negative controls and treated groups. Computerized densitometric analysis of the digital pictures obtained with the above-mentioned Leica DM5500 B fluorescence microscope was used to determine cell reactivity in all groups. ImageJ software (http://rsb.info.nih.gov/ij/) was used to calculate average densitometric values from the cell nucleus and cytoplasm. After removing the background in the regions of interest, the contrast index was calculated using the formula [(chosen area x 100)/total area] (three samples per group).

### *In vivo* study

Female C57BL/6J mice (6–8 weeks of age) were purchased from Fio Cruz Institute (Immunology Department, Recife, Pernambuco, Brazil). Mice were housed in an animal facility under a 12‐hr light/dark cycle and were given standard chow and water *ad libitum*. Mice weighing 18–22 g at 7–9 weeks of age were used for this study.

#### Isolation and characterization of peritoneal macrophages

Isolation and characterization of peritoneal macrophages from mice were described by Hunter et al [29]. Briefly, after the mice were killed, 5 ml of ice-cold PBS were injected into the abdomen. Immediately after injection, the fluid was withdrawn and centrifuged at 350 g for 5 min at 4°. Subsequently, the cell pellet was resuspended in 1 ml of Dulbecco's modified Eagle's medium supplemented with 2% penicillin-streptomycin and 10% bovine serum. Then, in Petri dishes, the peritoneal exudate was cultured for 24 hours at 37°. After this time, non-adherent cells were removed and adherent cells were kept to increase the density, changing the medium every two days for one week. Before the *in vitro* differentiation, macrophages were characterized by immunofluorescence for Anti-F4/80 (1:200, ABCAM, ab6640 , USA) [30].

#### *In vitro* differentiation of macrophages towards M2-TAM

The method used to differentiate the macrophages has been described previously by Hunter et al. (18). Briefly, M2‐polarized macrophages were differentiated from peritoneal macrophages by the addition of mouse recombinant IL‐4 (10 ng/ml; Invitrogen, Carlsbad, CA) for 48 hrs. Furthermore, peritoneal macrophages were treated with mouse recombinant IL‐4 (10 ng/ml) plus NP3 (RA)-CHO, abIL-10R, and NP3 (RA)-CHO+ abIL-10R for 48 hours to assess their effect in the polarization process.

#### Flow cytometry

After incubation and treatment with IL-4 and/or 2.5 μg/ml NP3 (RA)-CHO, 1.2 μg/ml abIL-10R, and 2.5 μg/ml NP3 (RA)-CHO+1.2 μg/ml abIL-10R for 48 hours, cells (1.5 × 10^4^ cells/well in a 12-well plate) were collected with a cell scraper and blocked with 0.5 % BSA in PBS for 45 minutes. Cells were labelled with anti-mouse CD206-PerCP antibody (1:1000) [11] to evaluate whether the cells showed characteristics of M2 macrophages. After a final washing step, the cells were analysed by flow cytometry.

### CRC allographic in vivo models and treatment regimens

A suspension of 1 × 10^6^ CT-26 cells was subcutaneously injected into the flank of male Balb/c mice to induce colorectal tumors [57]. The UFRN's Committee on the Ethics of Animal Experiments accepted the procedure (CEUA, Permit Number: 170.020/2019).

Tumour growth was monitored daily with calliper and upon reaching 3-4 mm in diameter, animals were categorized into six groups (N=5, each group) and peritumorally treated three times within 15 days with: (1) Control (untreated) = 5 mg/kg saline solution; (2) Oxaliplatin (OXA) = 5 mg/kg; NP3 (RA)-CHO = 1.25 mg/Kg; (4) 1.25 mg/kg saline solution, previously treated (30 minutes before) with anti-PD-L1 inhibitor = 10F.9G2, 2.16 mg/kg; (5) NP3 (RA)-CHO = 1.25 mg/kg, previously treated (30 minutes before) with anti-PD-L1 inhibitor (10F.9G2, 2.16 mg/kg); (6) NP1 (OXA, RA)-CHO25 = 1.25 mg/kg, previously treated (30 minutes before) with anti-PD-L1 inhibitor (10F.9G2, 2.16 mg/kg). Prior administration of the anti-PD-L1 inhibitor was only given in the first treatment, with the others being administered only saline solution, NP3 (RA)-CHO or NP1 (OXA, RA)-CHO. In this line, some mice were treated with OXA, a standard chemotherapy to CRC, and with NP1 loaded with OXA+RA and coated with CHO [57]. They were used in this study as control groups to compare their effects to NP3, which contained RA but not OXA. During treatment the diameter of the tumours was measured every two days. Five days after the last treatment (day 19), the animals were euthanized and blood was collected from the cardiac cavity (for biochemical analysis), as well as the tumour for signalling pathway analysis of tumour progression by quantitative real-time (qRT)-PCR and immunohistochemistry. Lungs and liver were collected for histopathological analysis of metastasis using haematoxylin-eosin (H&E) staining and immunohistochemistry. Results were expressed as a growth curve from the average tumour volume (mm³) calculated by the following equation according to Oliveira et al. [57]: volume = (width x length²) x 0.52.

#### *In vivo* imaging

When the size of mice tumour reached 3-4 mm in diameter, 1.25 mg/kg of NP3 (holding RA and IR-780 iodide coated with CHO) was injected via peritumour (mice=n 3). Images were taken at 2, 24 and 72 h after injection using the *in vivo* imaging system (Kodak In‐Vivo FX image station, USA). The excitation wavelength of IR-780 is 735 nm and emission spectrum of it is 780–900 nm.

#### mRNAExpression analysis

Total RNA was extracted from tumour tissue pieces using the SV Total RNA Isolation System (Promega, Madison, WI, USA) and the trizol reagent (Invitrogen Co., USA). Qrt-PCR analyses of β-actin, E-cadherin, vimentin, PDL-1, STAT-3, NF-kB, CD163, CD68, CD8, CD25, CCL22, and CXCR4 were carried out using the Applied Biosystems 7500 FAST system (Applied Biosystems, Foster City, CA, USA) together with a SYBR Green Mix and the primers according to a standard protocol for the primers listed in Table S1. The conventional PCR settings were 50°C for 2 minutes and 95°C for 10 minutes, followed by forty 30-second cycles at 94°C, 30 seconds at a variable annealing primer temperature, and 1 minute at 72°C. The relative expression levels of the target genes for the experimental groups were calculated using mean Ct values compared to those in the negative control group; expression data was normalized using the 2–ΔΔCt formula relative to the housekeeping gene β-actin [25].

#### Histopathological analysis: H&E and immunohistochemical staining

Tumours were fixed in 10% neutral buffered formalin, dehydrated, and embedded in paraffin. For H&E staining, 5-μm-thick sections were cut and were examined by light microscopy (10 × and 40 × magnification).

Using a microtome, 4-µm-thin tissue slices were cut from tumours and transferred to gelatine-coated slides. The paraffin‐embedded tumour tissue sections were incubated overnight at 4°C with anti‐STAT3 (Santa Cruz Biotechnology, USA), anti‐CD163 (Proteintech, USA), anti‐TGF‐β (Santa Cruz Biotechnology, USA), anti‐vimentin (Invitrogen, USA), anti‐IL‐10 (Invitrogen, USA), and anti-CXCL12 (Novusbio, USA) (all in a dilution to 1:300). Slices were rinsed in PBS and treated with a secondary antibody conjugated to streptavidin/Haptoglobin-Related Protein (HRP) (Biocare Medical, Concord, CA, USA). Immunoreactivity to various proteins was visualized using a colorimetric-based detection kit (TrekAvidin-HRP Label + Kit from Biocare Medical, Pacheco, USA) according to the manufacturer's procedure. The digital images were captured using a high-power objective (40 ×) and a light microscope (Nikon Eclipse 2000 equipped with Nikon DS-Fi2; Nikon Corporation, Tokyo, Japan). The following scale was used to as-sess the intensity of cell immunostaining: Absence of positive cells (#1), small number of positive cells (#2), moderate number of positive cells (#3), and substantial number of positive cells (#4). Two previously trained examiners performed a double-blind evaluation of labelling intensity [18].

### Primary CRC tissue microarray

Biopsies were taken from 180 patients at the Cancer Referral Center of Natal, Brazil, who were undergoing surgical resection of CRC. Additional clinical data, as well as the process for creating tissue microarray (TMA) blocks, were previously described [26]. The institutional committee approved this study (No. 030/0030/2006).

Anti-E-cadherin, anti-STAT3, anti-NF-Κb, anti-PD-L1, anti-CXCL12, and anti-CD163 antibodies were used at a dilution of 1:1500 for immunohistochemistry (All Proteintech, Manchester, UK). A light microscope with a high-power magnification (40 ×) was used to count the number of positive cells within each TMA core. Two researchers were blinded to the clinical data and independently analysed E-cadherin, β-catenin, galectin-3, PD-L1, CXCL12, and CD163 expression in the tumour and surrounding stromal tissue. The modified Dukes’ criterion classification was used to stage the disease [31]. The cytoplasmic staining was assigned to one of four groups: 1: no detectable staining or staining less than 10 % of the total sample; 2: weak but detectable staining or staining between 10 % and 25 % of the total sample; 3: moderate staining, clearly positive but still weak or staining between 25 % and 75 % of the total sample; and 4: intense staining or staining greater than 75 % of the total sample. For all statis-tical calculations, this grading system was used. A Nikon light microscope at 400 × magnification was used to undertake a blinded semiquantitative evaluation of stained TMAs [31].

### Statistical analysis

All data are presented as mean ± standard deviation (SD). Comparisons were made with Student's t-test or ANOVA (non-parametric) with Bonferroni or Dunnet tests . All experiments were replicated two or three times, with similar results. Using Kaplan-Meier product limit survival curves with a log-rank (Mantel-Cox) comparison test, the likelihood of survival over time compared to immunohistochemical labelling antibodies for E-cadherin, β-catenin, galectin-3, PD-L1, CD163, and CXCL12 was assessed. The length of survival was calculated from the time of diagnosis to the time of death. Values of p<0.05 were considered statistically significant in all tests.

## Results

### Properties of PLGA NPs and internalization of nanoparticles

**Fig. 1:**
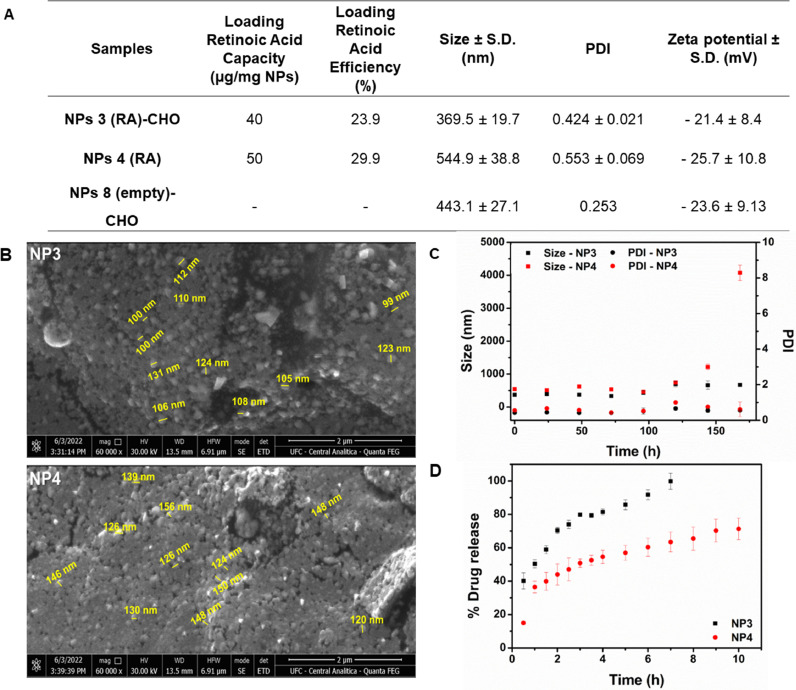
Characterization of Nanoparticles. (A) Physicochemical properties of Poly(D,L-Lactide-co-GlycolicAcid)(PLGA) nanoparticles(NPs). Determination of retinoic acid (RA) content, size distribution, and zeta-potential of PLGA NPs. Particles were characterised by Dynamic Light Scattering (DLS) and zeta-potential measurements. Particle size data represent the mean value ± standard deviation (SD) of dynamic light scattering data. Zeta-potential data represent the mean value ± SD of 10 readings. RA contents of PLGA NPs were determined by particle digestion and measured by reversed-phase high-performance liquid chromatography (RP-HPLC) analysis. (Polydispersity Index). (B) Morphology images of NP3 and NP4 obtained by SEM. (B), Stability of size and PDI as function of time (C) . Cumulative release of RA in the first 10 h (D).

We loaded PLGA NPs with RA (NP3, NP4) and coated the NPs with CHO (NP3). NP8 was coated with CHO, but did not contain RA. The final formulation of our NPs was NP3-(RA)-CHO, NP4-(RA) and NP8-CHO. The PLGA NPs were first characterized in order to determine their size and surface charge (Fig. 1A). The average diameter of the particles ranged from 369.5 nm to 544,9 nm. The average zeta potential ranged from -23.6 mV to -28.5 mV. The MEV investigation of PLGA NPs revealed that all PLGA NPs were spherical in shape with a uniform size distribution, as shown in Fig. 1 B. The average particle size obtained by SEM were 111.2 ± 10,7 nm and 137.8± 12.7 nm respectively for NP3 and NP4. The size of these particles was smaller than the ones obtained by DLS and it can be due to the fact of SEM analysis were caried out with dry sample while for DLS the measure were done in solution. The stock stability of colloidal dispersion showed be stable up to 5 days, with no significant variation of size and polydispersion index (Fig. 1C). However, NP4 colloidal dispersion aggregated after 6 days with an increase in size from 544.9 (first day) to 4075 nm. The PLGA-RA system showed a slow, gradual and progressive release (Fig. 1D). Based on this result, the release assay of RA from NP3-(RA)-CHO and NP4-(RA) in PBS initially showed different patterns with about 100% of RA released around 8 hours for NP3-(RA)-CHO and 60% of RA for the NP4-(RA) (Fig. 1D). RAW 264.7 cells and CT-26 cells were more able to internalize NP3 than NP4 within 4 hours and 24 hours (Fig.1S).

### Low doses of RA, CHO, NP3-(RA)-CHO, NP4-(RA), NP8-CHO and IL-10R blocking antibody (ABIL-10R) did not affect cell viability

**Fig. 2:**
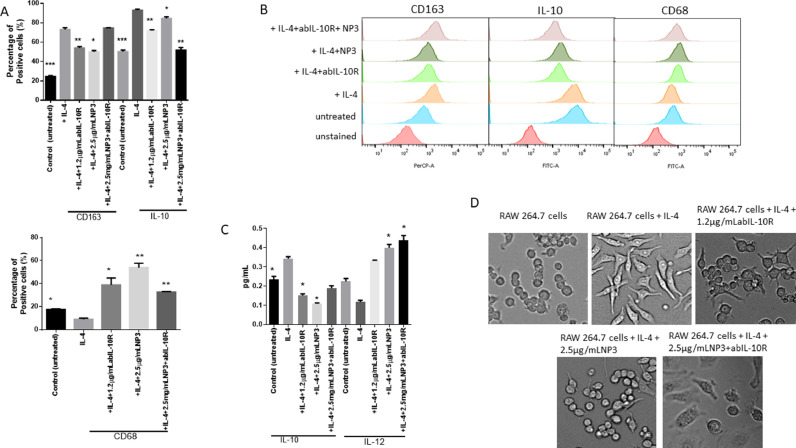
NP3 and anti-IL-10R impair M2-TAM polarization. (A) Percentage of cells positive for the M2 markers CD163 and IL-10, and the M1 marker CD68 on IL-4-polarized RAW 264.7 cells (M2-TAM), in the presence and absence of 2.5 μg/mL NP3 or 1.2μg/mL abIL-10R after 48 hours of polarization. (B) Representative flow cytometry profiles of CD163, IL-10, and CD68 expression of cultured RAW 264.7 cells as well as after treatment with IL-4, or IL-4, and NP3 and/or anti-IL-10R. (C) ELISA analysis of the M1- and M2-related cytokines IL-10 and IL-12, respectively, from cell culture supernatants of IL-4-polarized RAW 264.7 cells, in the presence and absence of 2.5 μg/mL NP3 or 1.2μg/mL abIL-10R. All p-values were compared to RAW 264.7cells+IL-4, ****p<0.001. The data represent the mean±SEM of 3-6 independent experiments. All p-values were compared to control cells by analysis of variance and Dunnet's test. (D) Representative phase-contrast images of control and IL-4 polarized RAW 264.7 cells, in the presence or absence of 2.5 μg/mL NP3 or 1.2μg/mL abIL-10R.

We first investigated whether RA, CHO, NP3, NP4, NP8, and abIL-10R caused cytotoxicity in CT-26 and RAW 264.7 cells in a concentration-dependent manner (Fig.S2A-H). The main goal of this experiment was to analyse the ideal non-citotoxicity concentration of compounds and nanoparticles to perform following assays of polarization, cell migration, EMT and cell proliferation. The viability of CT-26 cells decreased by 7.33 % and 10.67 %, respectively, when the cells were incubated with 2 μg/ml RA for 24 hours and 48 hours. The viability of CT-26 cells further decreased to 9.33% and 19 % with 10 μg/ml CHO for 24 hours and 48 hours, respectively (Fig.S2A-B). Similar to RA and CHO, 5 μg/ml NP3, NP4 and NP8 reduced CT-26 cell viability by 7.33% to 10.33 % and 22 % to 26.67% after incubation for 24 hours and 48 hours, respectively (Fig.2SC-D), which is in accordance with our results from the other two NPs. Furthermore, the cell viability decreased by 8.67 % and 19 %, respectively, after 24 hours and 48 hours incubation with 2.4 μg/ml abIL-10R (Fig.S2C-D).

However, RA applied at 2 μg/ml reduced the viability of RAW 264.7 cells by only 9.33 % and 11.33 % after incubation for 24 hours and 48 hours, respectively (Fig.S2E), and 10 μg/ml CHO to a maximum of 12.67 % after 48 hours of incubation (Fig.S2F). In addition, 5 μg/ml NP3, NP4, and NP8 decreased the viability of RAW 264.7 cells by 10.33%, 9.33% and 7.33%, respectively (Fig.S2 G-H). In 48 hours, NP3, NP4 and NP8 decreased the viability by 16.33%, 17.67% and 12.33%, respectively (Fig.S2G-H). Furthermore, 2.4 μg/ml abIL-10R decreased the cell viability of RAW 264.7 cells by 8.76% and 14.67% after incubation for 24 hours and 48 hours, respectively (Fig.S2G-H).

### NP3, ABIL-10R, and a NP3+ABIL-10R combination attenuated M2-TAM polarisation

In order to assess whether RA, CHO, NP3, NP4, NP8, and abIL-10R can modify the polarisation of TAMs, we polarised RAW 264.7 macrophages towards M2-TAMs in the presence of IL-4 for 48 hours. Based on non-citoxicity concentrations, M2-TAMs were cultured in the presence or absence of 1 μg/ml RA, 5μg/ml CHO, 2.5 μg/ml NP3, 2.5 μg/ml NP4, NP8 or 1.2 μg/ml abIL-10R (concentration without cell cytotoxicity to either cell line). Next, the expression of M1- and M2-related markers was analysed in IL-4-stimulated RAW 264.7 cells by flow cytometry to characterise these macrophages. Flow cytometric analysis showed that expression of the M2-markers IL-10 and CD163 was significantly higher than in control RAW 264.7cells (Fig. 2 A+B; both p<0.0001), demonstrating the successfully modulation of classical macrophages to M2-TAMs using IL-4. In addition, CD163 (Fig. 2 A+B, p<0.001 and p<0.05) and IL-10 (Fig. 2A; p<0.001, p<0.05, and p<0.001) expression significantly decreased after treatment with 1.2 μg/ml anti-IL-10R inhibitor, 2.5 μg/ml NP3, and 2.5 μg/ml NP3+1.2 μg/ml abIL-10R. In addition, the expression of the M1 marker CD68 was reduced in M2-TAMs (Fig. 2A+B) and the expression was significantly increased after adding 1.2 μg/ml abIL-10R, 2.5 μg/ml NP3, and 2.5 μg/ml NP3+1.2 μg/ml abIL-10R during the M2 polarisation (Fig. 2A+D; p<0.05, p<0.001 and p<0.001, respectively). Furthermore, the expression of CD163 (p<0.05) decreased after treatment with 1 μg/ml RA, but not IL-10 expression (Fig.S3A). On the other hand, CD68 expression (p<0.05) increased in the presence of 1 μg/ml RA (Fig.S3B). However, expression of the polarisation-related markers (CD163, IL-10, and CD68) was not significantly altered in RAW 264.7 cells by means of of 5 μg/ml CHO, 2.5 μg/ml NP4, and 2.5 μg/ml NP8 (Fig.S3A). Based on these results, NP3 showed higher efficiency in blocking M2 polarisation and stimulating the switch to M1 macrophages than RA, free CHO, NP4 and NP8. These results showed that the advantage of using the nanoparticle coated with CHO and holding RA (NP3) over free RA or a nanoparticle loaded with RA but without CHO (NP4) is due to the blockage of polarization towards M2  and the stimulation to M1 more effectively.

**Fig. 3:**
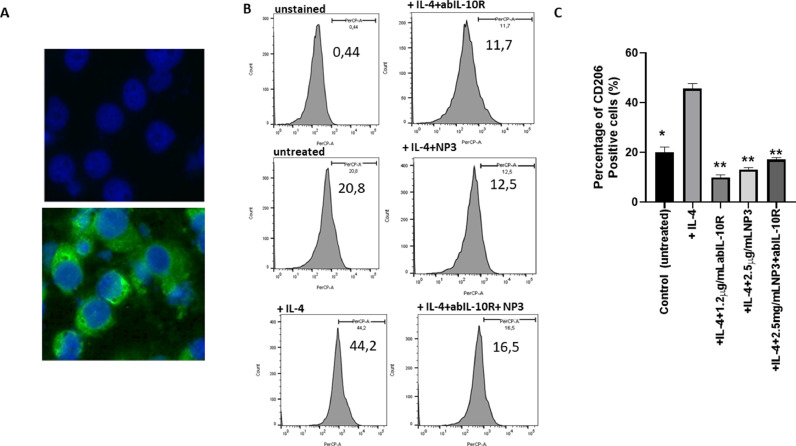
Profile of Peritoneal-derived macrophages stimulated with IL-4, NP3 and abIL-10R. A). Immunofluorescence assay for the expression of F4/80 in isolated peritoneal-derived macrophages . B) Representative flow cytometry profiles of CD206 expression of peritoneal derivated-M1 macrophage after treatment with IL-4, or IL-4, and NP3 and/or anti-IL-10R. C) Percentage of cells positive for the M2 markers CD206, on IL-4-polarized peritoneal-derived macrophages (M2-TAM), in the presence and absence of 2.5 μg/mL NP3 or 1.2μg/mL abIL-10R after 48 hours of polarization. The cell nuclei were stained with DAPI (blue; scale bar=200 µm). All p-values were compared to IL-4 polarized peritoneal-derived macrophages. by analysis of variance and the Dunnett's test (**p<0.01, *p<0.05).

Therefore, all of the following experiments were performed with NP3. Furthermore, these results suggest that the effect of NP3 in combination with abIL-10R was not stable because both of them acted at the same target sites of down-stream signalling what provoked a saturation in downregulating M2-polarization. These findings were confirmed using isolated peritoneal macrophages induced to M2 -TAM by IL-4 polarization. The CD206 expression of M2 marker increased significantly after using IL-4. On the other hand, the expression decreased after adding 1.2 μg/ml abIL-10R, 2.5 μg/ml NP3, and 2.5 μg/ml NP3+1.2 μg/ml abIL-10R during the M2 polarisation (Fig. 3*B-C; all p<0.001).*

*To further characterize the macrophages, we collected the supernatant of the cell culture and measured the amounts of M2- and M1-related cytokines IL-10 and IL-12, respectively, by ELISA (* Fig. 2 *C).*

M1- and M2-TAMs were morphologically distinct from each other, as observed by phase-contrast microscopy (Fig. 2D). When compared to the other two populations, M2-TAMs were more elongated, having spindle-like cytoplasmic extensions at the cell poles. After incubation with IL-4 and treatment with abIL-10R, NP3, and NP3+abIL-10R, the cells lost some of their M2-TAM morphological characteristics and started resembling M1 and naive RAW 264.7 cells (Fig. 2D).

### IL-10 showed pro-proliferative properties in CRC cells

**Fig. 4:**
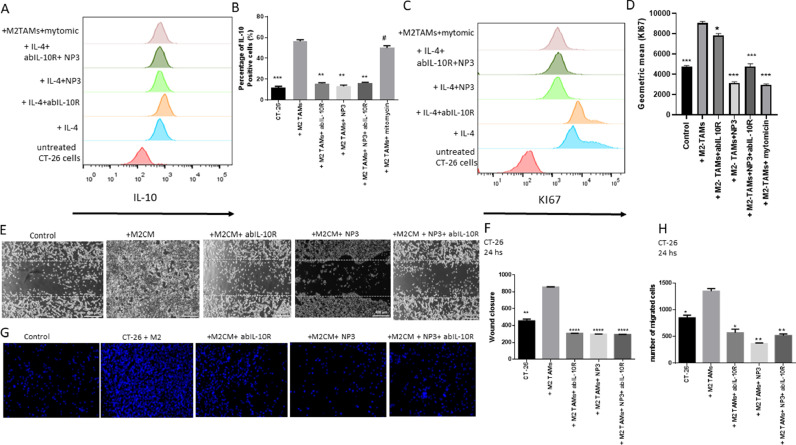
CT-26 cells co-cultured with NP3 and/or abIL-10R-treated M2-TAMs in a transwell system showed decreased expression of IL-10 and cell proliferation. (A) Representative flow cytometry profiles of IL-10 expression on control CT-26 cells and after co-culture with IL-4, or IL-4 and 2.5 μg/mL NP3 and/or 1.2μg/mL abIL-10R-treated RAW 264.7 cells. (B) Flow cytometric analysis of the mean fluorescence intensity of the M2 marker IL-10 in control CT-26 cells and after co-culture with IL-4, or 2.5 μg/ml NP3 and/or 1.2μg/ml abIL-10R-treated RAW 264.7 cells. (C) Representative flow cytometry profiles and (D) quantification of the mean fluorescence intensity of Ki-67 expression in control CT-26 cells and after co-culture with IL-4, or 2.5 μg/ml NP3 and/or 1.2μg/ml abIL-10R-treated RAW 264.7 cells. (E) Migratory properties of CT-26 cells after co-culture with M2-TAM-CM were assessed in a wound-healing assay. Phase-contrast images of CT-26 cells after 24 hours. CT-26 cells were cultured in the presence of M2-TAM-CM, with CM of M2-TAMs treated with 2.5 μg/ml NP3 and/or 1.2μg/ml abIL-10R-treated RAW 264.7 cells and the proliferation-inhibitor mitomycin C. The dotted lines represent the wound (scratched) area (scale bar=400 µm). MMC=Mitomycin C. (F) Quantification of CRC cells that migrated into the wound area (area between the dotted lines) after 24 hours. All p-values were compared to CT-26 cells co-cultured with CM of IL-4-treated RAW 264.7 cells by analysis of variance and the Dunnett's test. (G) Fluorescence microscopy images of transmigrated CT-26 cells. CT-26 cells were co-cultured with M2-TAMs in a transwell system for 24 hours, in the presence or absence of 2.5 μg/ml NP3 or 1.2μg/ml abIL-10R. The cell nuclei of transmigrated cells at the bottom of the transwell insert were stained with DAPI (blue) and (H) quantified using Fiji image analysis software (see Materials and methods; scale bar=25 µm; *p<0.05; **p<0.01; ***p<0.001, ***p<0.0001 versus CT-26 and MC38 cells in co-culture with M2-TAMs or M2-TAM-CM).

The expression of IL-10 in CT-26 cells was reduced by the addition of NP3-treated M2-TAMs (Fig. 4 *A*+ *B*). Next, we looked whether M2-TAMs and CRC cells might form an immunosuppressive TME, and whether NP3 could prevent this interaction. Thus, CT-26 cells were co-cultured with NP3-treated M2-TAMs in the presence of an abIL-10R in a transwell system. Flow cytometry was used to examine the expression of intracellular IL-10 in CT-26 cells after 24 hours (Fig. 4A-B). CT-26 cells expressed basic IL-10 without treatment. However, when co-cultured with M2-TAMs, intracellular IL-10 levels in CT-26 cells increased considerably, which was inhibited by adding the IL-10R blocking antibody. Treatment of M2-TAMs with NP3 reduced IL-10 expression in CT-26 (p<0.01, Fig. 4A+B). In addition, co-culture with IL-10R blocking antibody and NP3-treated M2-TAMs further reduced IL-10 expression (p<0.01, Fig. 4 A+B). In summary, these results suggest that NP3 suppresses IL-10 expression of M2-TAMs in CRC cells upon co-culture.>

Next, we investigated whether reduced levels of IL-10 in CT-26 cells could influence cellular functions, such as cell proliferation, in a transwell system after co-culture with IL-10R blocking antibody together with NP3 or NP3-treated M2-TAMs. Thus, we assessed the percentage of proliferating cells by means of Ki-67 labelling. Flow cytometry was used to examine CT-26 cells after 24 hours (Fig. 4C+D). The amount of proliferation marker Ki-67-positive cells was doubled when CT-26 cells were indirectly co-cultured with M2-TAMs. This impact was mediated by IL-10, since inhibiting IL-10R lowered Ki-67 expression levels below the basal levels obtained in CT-26 cells cultured in the absence of M2-TAMs. Surprisingly, NP3 was just as effective in inhibiting proliferation as the proliferation inhibitor mitomycin C. In conclusion, IL-10 showed pro-proliferative effects on CRC cells, which was completely abolished by addition of NP3 and the combination of NP3 and IL-10R blocking antibody during the co-culture.

### NP3, ABIL-10R, and a NP3+ABIL-10R combination suppressed M2-TAM-induced migratory and invasive properties in CT-26 cells

We wanted to see if NP3, anti-IL-10R inhibitors, or a combination of NP3 and anti-IL-10R inhibitor would prevent CT-26 cells from undergoing M2-TAM-induced EMT. CT-26 cells were cultivated in the presence or absence of M2-TAMs to see if M2-TAMs had a direct impact on CRC cell motility. M2-TAMs stimulated migration of CT-26 cells compared to non-treated cells (Fig. 4G+H; p<0.05). This effect could be blocked by adding NP3 (p<0.001), abIL-10R (p<0.05), and NP3+abIL-10R (p<0.001) during co-culture (Fig. 4G+H), suggesting that the treatment inhibits the process by which M2-TAMs induce migration.

Next, we examined whether NP3, abIL-10R, or a combination of NP3+abIL-10R could inhibit CT-26 cells from migration by either directly affecting the cancer cells or via M2-TAMs. As a result, we used a wound healing assay to measure the migratory capabilities of CT-26 cells. M2-TAMs were cultivated in the presence or absence of NP3, abIL-10R, and the NP3+abIL-10R combination. Cells were treated with CM of M2-TAMs. During this experiment, we treated CT-26 cells with mitomycin C to ensure that we could detect cell migration and proliferation in the wound area. Next, we observed their migration into the wound area after 24 hours (p<0.001; Fig. 4E+F). Significantly fewer CT-26 cells migrated into the wound area when treated with M2-TAM-CM as well as after treatment with NP3, abIL-10R, and NP3+abIL-10R (all p<0.0001, 24 hours, Fig. 4E+F).

In conclusion, our findings show that M2-TAMs induce EMT and cell mobility in CRC cells. In addition, we were able to demonstrate that NP3, abIL-10R, and the NP3+abIL-10R combination reduce these processes. Moreover, our findings show that M2-TAM-CM factors restrict cancer cell migration.

### NP3, ABIL-10R, and the NP3+ABIL-10R combination decreased the mesenchymal phenotype of CRC cells

**Fig. 5:**
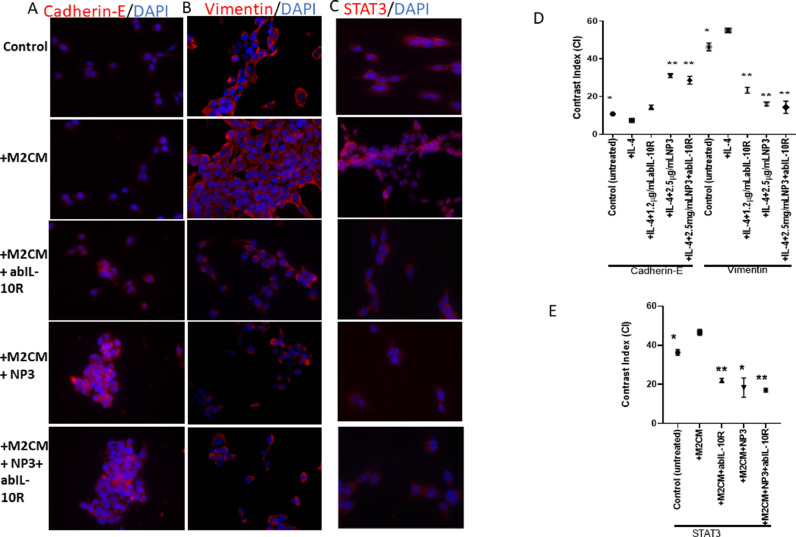
Co-culture of CT-26 cells with M2-TAMs increased the mesenchymal phenotype in CRC cells. CT-26 cells were co-cultured with CM of 2.5 μg/ml NP3 and/or 1.2μg/ml abIL-10R-treated M2-TAMs for 48 hours and analysed by fluorescence microscopy for expression of (A) E-cadherin, (B) vimentin, (C) STAT-3. The cell nuclei were stained with DAPI (blue; scale bar=25 µm). (D) Quantification of the fluorescence intensity of the E-cadherin and vimentin labelling in CT-26 cells after co-culture with CM from NP3 and/or abIL-10R-treated M2-TAMs. (E) Quantification of the fluorescence intensity of the STAT3 labelling in CT-26 cells after co-culture with CM from NP3 and/or abIL-10R -treated M2-TAMs for 48 hours. All p-values were compared to CT-26 cells co-cultured with CM from IL-4-treated RAW 264 by analysis of variance and the Dunnett's test (**p<0.01, ***p<0.001 versus M2-TAM-CM or M2-TAM).

Tumour cells undergoing EMT have distinct phenotypic alterations that are linked to their increased migratory abilities. Thus, we investigated whether NP3, anti-IL-10R inhibitor, and the NP3+anti-IL-10R inhibitor combination are able to suppress cell mobility. CT-26 cells were cultured with M2-TAM-CM and the expression of the epithelial marker E-cadherin, the mesenchymal marker vimentin, as well as the proliferation and survival marker STAT3 were analysed using immunofluorescence (Fig. 5A-E). CT-26 cells cultured with or without M2-TAM-CM showed low expression of E-cadherin. However, the expression of E-cadherin significantly increased in CT-26 cells when cultured with M2-TAMs -CM + NP3, and NP3+abIL-10R (both, p<0.001,). However, the expression of vimentin and STAT3 was high in non-treated CT-26 cells, indicating their mesenchymal phenotype, which is associated with migration, invasion, proliferation, and survival. Upon adding M2-TAM-CM to CT-26 cells, vimentin and STAT3 expression increased significantly. However, the expression of vimentin and STAT3decreased significantly when CT-26 cells were treated with M2-TAM+NP3, abIL-10R, and the NP3+abIL-10R (Fig. 5B+D, vimentin, all p<0.001; Fig. 5C+E, STAT3, p<0.001, p<0.05, and p<0.001, respectively;). Our data indicate that NP3, abIL-10R, and the NP3+abIL-10R combination suppress cell mobility, proliferation, and survival in CT-26 cells, which was mediated by our M2-TAM-CMs.

### *In vivo* study

**Fig. 6:**
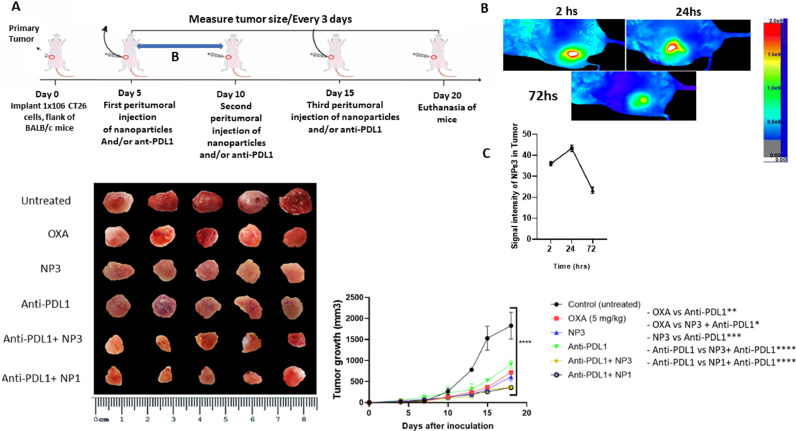
Therapeutic scheme, inhibition of tumour growth and In vivo fluorescence imaging.The growth curves of colorectal tumours in BALB/c mice show a reduction in tumour volumes after treatment with 5 mg/kg free OXA; 1.25 mg/kg CHO-coated, RA-loaded PLGA NP3; 2.16 mg/kg anti-PD-L1 inhibitor; 2.16 mg/kg anti-PD-L1 in-hibitor+1.25 mg/kg CHO-coated, RA-loaded PLGA NP3; 2.16 mg/kg anti-PD-L1+1.25 mg/kg CHO-coated, OXA-RA-loaded PLGA NP1 (A). The excised tumours were presented on a panel for comparative analysis of tumour size.  In vivo  imaging (B) and signal intensities of 1.25 mg/kg of NP3 (holding RA and IR-780 iodide coated with CHO) injected in CT26 tumor in mice at 2, 24 and 72 h post-injection, respectively (C); One-way ANOVA with post-hoc Bonferroni correction or Kruskal-Wallis with post-hoc Dunn correction were performed (*p<0.05, **p<0.01, ***p<0.001, ****p<0.0001). All data are presented as mean±SD of five independent values (n=5) per group, except to experiment of in vivo fluorescence imaging (n=3).

First of all, we wanted to study the immune profile in the primary tumour comparing the effect of NP3 alone or in combination with anti-PD-L1 immune check-point blocker. Based on in previous study of our group [18], we showed that PD-L1 protein has a strong immunosuppressive effect under TME by down-regulating IL-10 and TGF-β via M2-TAM in breast cancer. In this study, tumours, which were previously treated with anti-PD-L1 inhibitor in combination with NP3 showed a higher anti-tumour efficiency than free OXA and NP3. The tumour growth curve reveals that different PLGA formulations promoted a significant reduction of tumour volume (p<0.0001, NP1 and NP3 including anti-PD-L1 pre-treatment) of ∼30% and ∼70% when compared to free OXA and saline-treated animals, respectively (Fig. 6). Interestingly, there was statistical difference between the treated groups, mainly when compared to the anti-PD-L1 inhibitor (p<0.001, OXA × anti-PD-L1; p<0.005, NP3 × anti-PD-L1; p<0.0001, an-ti-PD-L1-NP3/NP1 × anti-PD-L1). However, no significant differences were observed between NP1 and NP3 in combination with the pre-treatment with anti-PD-L1 inhibitor (p>0,05; Fig. 6). These results suggest that anti-PD-L1 inhibitor potentiated the anti-tumour effect of RA-loaded, CHO-coated NPs, especially without the necessity of chemotherapy (OXA).

### NP3 downregulated TAM-modulated EMT and metastasis and upregulated the immune response in primary and metastatic tumours

**Fig. 7:**
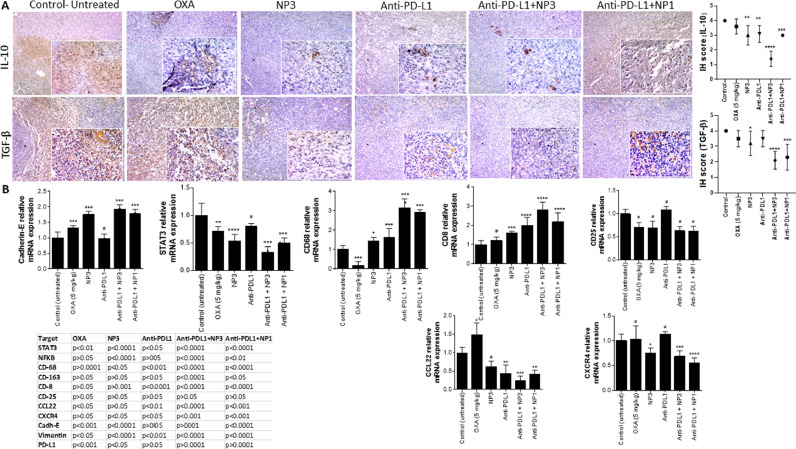
Expression profiles of colorectal tumours after different treatments. Treatment with NP3 alone or previously with anti-PD-L1 reduced EMT ( E-Cad), immunosuppression ( IL-10, TGF-β,  CD68,  CD8,  CD25), and metastasis factors  CCL22,  CXCR4) by down-regulating the STAT3/NF-κB pathway (in the primary TME. The tumours excised from the previously treated female BALB/c were analysed for immunohistochemistry (A) and relative gene expression (B). One-way ANOVA with post-hoc Bonferroni correction or Kruskal-Wallis with post-hoc Dunn correction was performed (*p<0.05; **p<0.01, ***p<0.001, ****p<0.0001). All data are presented as mean±SD of five independent values (n=5) per group with at least two replicates.

**Fig. 8:**
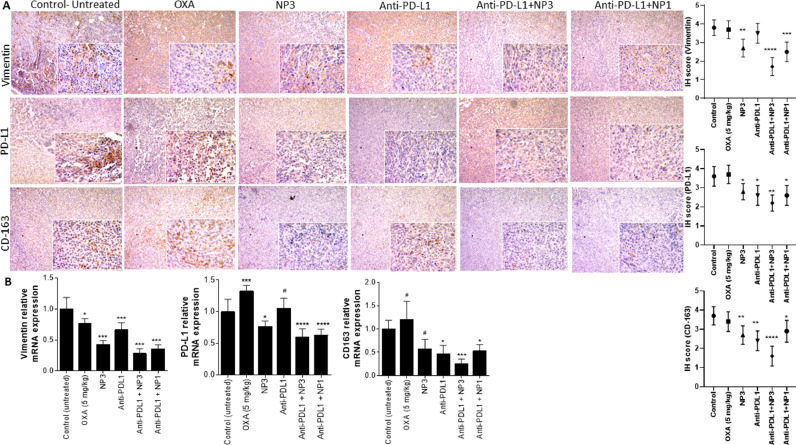
Expression profiles of colorectal tumours after different treatments. Treatment with NP3 alone or previously with anti-PD-L1 reduced EMT ( vimentin) and immunosuppression ( CXCL12) by down-regulating the STAT3/NF-κB pathway (via M2-TAM ( CD163) in the primary TME. The tumours excised from the previously treated female BALB/c were analysed for immunohistochemistry (A) and relative gene expression (B). One-way ANOVA with post-hoc Bonferroni correction or Kruskal-Wallis with post-hoc Dunn correction was performed (*p<0.05; **p<0.01, ***p<0.001, ****p<0.0001). All data are presented as mean±SD of five independent values (n=5) per group with at least two replicates.

In the TME, TAMs influence several signalling cascades, such as EMT, and consequently metastasis. Therefore, we investigated the expression of CD163+ TAMs, CD68+ M1s, CD8+ lymphocytes, and PD-L1 and CD25+ Treg cells within the primary tumours. In addition, EMT was studied by analysing the expression of E-cadherin and vimentin. After 15 days of treatment, E-cadherin, CD68, and CD8 were increased, while vimentin, STAT3, NF-κB, CD163, and CD25 were reduced (as detailed in Table Expression, Figs. 7 A,B and 8 A,B) in tumours of Balb/c mice when compared to the control group. Interestingly, anti-PD-L1+ NP3 treatment decreased the CD163 expression of M2-TAMs and significantly increase the expression of anti-tumour M1 macrophages and cytotoxic CD8 lymphocytes.

Our data indicates that targeting of metastasis factors in primary tumours is an effective management strategy for cancer and metastasis. Next, we investigated the expression of the two metastasis-related chemokines CCL22 and CXCR4, which are involved in seed-soil metastasis. NP3 decreased the expression of CXCL12, CCL22 and CXCR4 with or without anti-PD-L1 (Figs. 7 B and 8A). However, NP3 was more effective after anti-PD-L1 pre-treatment. To order to evaluated the expression of immunosuppressive cytokines such as PD-L1, TGF-β and IL-10 in TME after 15 days of treatment, we observed that their expression significantly decreased in the anti-PD-L1+ NP3 group when compared to other groups (Figs. 7 A,B and 8 B).

### Expression of proteins related to EMT, immunomodulation, and metastasis in human primary colorectal tumours

**Fig. 9:**
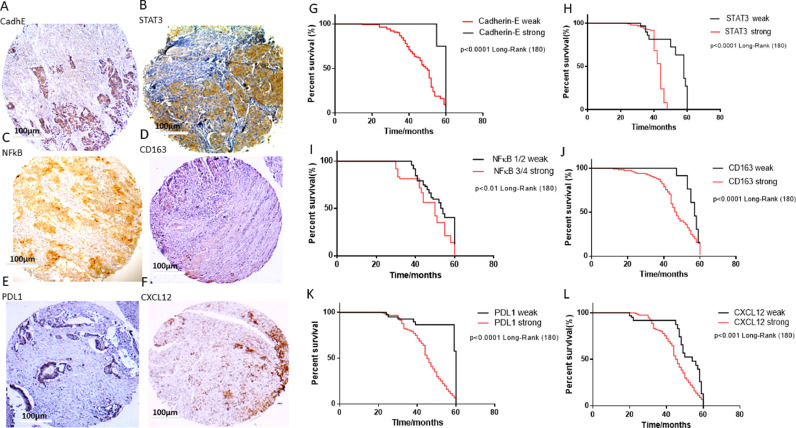
Expression of E-cadherin, STAT3, NF-κB, CD163, PD-L1, and CXCL12 in primary colorectal tumours by immunohistochemical staining. Colorectal adenocarcinoma with a high modified Dukes’ classification (“3” and “4”) showing: (A) weak E-cadherin cytoplasmic staining and (B-F) strong cytoplasmic staining of STAT3, NF-κB, CD163, PD-L1, and CXCL12, Magnification: 40X. (G) Kaplan–Meier survival curve estimated by E-cadherin staining in CRC tumours (log-rank x2=15.81; p<0.0001), (H) Kaplan-Meier survival curve estimated by STAT3 staining in CRC tumours (log-rank x2=30.45; p<0.0001), (I) Kaplan-Meier survival curve estimated by NF-κB staining in CRC tumours (log-rank x2=6.53; p<0.01), (J) Kaplan-Meier survival curve estimated by CD163 staining in CRC tumours (log-rank x2=17.72; p<0.0001), (K) Kaplan-Meier survival curve estimated by PD-L1 staining in CRC tumours (log-rank x2=25.23; p<0.0001), (L) Kaplan-Meier survival curve estimated by CXCL12 staining in CRC tumours (log-rank x2=8.05; p<0.001).

Herein, we evaluated if the expression of E-cadherin, STAT3, NF-κB, CD163, PD-L1, and CXC12 would be associated with a poor prognosis for patients with tumours with low (1/2) and high (3/4) modified Dukes’ classification in primary CRC. Low expression of E-cadherin was an indication of lymph node involvement (both, p<0.0001, Table S2), modified Dukes’ criteria grade (p<0.05, Table S2), and indicated a greater tendency towards death within 60 months of follow-up (p<0.0001 and p<0.01, respectively, Fig. 9A, C, G, I). In addition, patients with high expression of STAT3 and NF-κB were more likely to die within 60 months (both, p<0.0001, Table S2), increased lymph node involvement (both, p<0.0001, Table S2), and modified Dukes’ criteria grade (p<0.0001 STAT3, Table S3). Furthermore, high expression of CD63 and PD-L1 was associated with more aggressive tumours, as represented by lymph node involvement (p<0.0001 for both, Table S3), modified Dukes’ criteria grade (both, p<0.0001, Table S3), and an increased probability to die within 60 months (both, p<0.0001 Fig. 9D, E, J, K). Moreover, lymph node involvement (p<0.05, Table S4), modified Dukes’ criteria grade (p<0.01, Table S3), and an increased probability to die within 60 months (p<0.0001 Fig. 9F+L) was associated with high expression of CXCL12.

## Discussion and conclusions

Our study shows that the combination of anti-PD-L1 inhibitor and RA-loaded, CHO-coated NP3 exerts its anti-tumour effects by blocking the polarisation of M2-TAMs and, consequently the downregulating of EMT and immunosuppression in CRC. First, we revealed that treatment with RA-CHO-NP3 attenuated macrophage polarisation towards the M2 phenotype by suppressing the expression of the M2-related cytokine IL-10. In addition, we showed that RA-CHO-NP3 reduced the expression of PD-L1 and TGF-β in tumours as well as potentialized the anti-tumour activity of the anti-PD-L1 immune checkpoint blocker by downregulating M2-macrophage-mediated EMT and metastasis factors, and upregulating the cytotoxic activity of CD8+ lymphocytes in the TME. These findings supported our strategy of targeting M2-TAMs in the TME given that immunosuppressive phenotype of M2-TAMs and the upregulation of the STAT3/NF-κB signalling pathway crosstalk. In addition, the STAT3/NF-κB signalling pathway is involved in the formation of metastasis and, consequently, a poor treatment outcome of patients with CRC, as we found in this study. Our results identified RA-CHO-NP3 together with anti-PD-L1 immune checkpoint blocker as a promising agent to target M2 macrophages for future anti-metastasis therapy of CRC, especially for downregulation of the STAT3/NF-κB pathway via IL-10/PDL-1/TGF-β in the primary TME.

Tumour progression involves several processes related to cancer cells and the TME stroma [32]. However, immune factors related to tumours, such as IL-10 and IL-4, inhibit the production of pro-inflammatory cytokines and create immunosuppressive TAMs, which supports tumour growth [33]. Therefore, the TME has a high influence on the phenotype and function of macrophages [23] either by cell-cell interaction, cell cytokines or cell metabolites [23,34,35].

Consistent with previous reports [36,37], our data showed that the RA-loaded, CHO-coated NP3 attenuated the M2-TAMs. In addition, RA inhibited the expression of the anti-inflammatory cytokine IL-10 in M2-TAMs, and stimulated the switch to M1-TAMs [23]. Importantly, our in vitro study showed that NP3 was more effective in blocking M2-TAMs than free RA or NP4 (holding RA but no coated with CHO). These findings may be reinforced by the higher uptake of NP3 when compared to NP4 in the cellular uptake assay. This indicates that our NPs coated with CHO triggered a macrophage precise target via process of receptor-mediated endocytosis [38]. CHO coating of NPs enhanced their membrane fusion capability and, thus, improvement the drug delivery efficacy [39,40] by downregulating the cholesterol efflux and blocking retinoid X receptors in M2-TAM, moving them towards to M1 polarizing signals [24]. Strictly, tumour macrophages (M2-TAM) have a high expression of Retinoid X receptor beta (RXRB) on cell surface, despite to RXRB is known as a nuclear receptor [41]. Targeting RXRB seems to be a great strategy to downregulate M2-TAM. In our study, RA-loaded, CHO-coated NP3 prevents colorectal cancer cell migration via blocking the M2 polarization of TAMs. Furthermore, it is worth noting that NP3 had a similar effect as the anti-IL-10R inhibitor, meaning that IL-10-dependent polarisation of macrophages was attenuated by NP3. In the line with our findings, various studies found that the IL-10R/IL-10 axis is involved in overstimulation of the STAT3 pathway as well as downregulation of the NF-κB signalling pathway, which supports the M2-TAM phenotype [11,18,[42], [43], [44]].

For successful cancer management, finding early metastasis or, better, preventing EMT, including migration and infiltration of cancer cells is of utmost importance [45]. Our treatment with NP3 *in vitro*, with or without abIL-10R, was able to downregulate the expression of vimentin, thereby, reducing migration and EMT as well as upregulating the expression of E-cadherin. E-cadherin is poorly expressed in aggressive colorectal tumours, whereas vimentin has a high expression due to the migratory capacity acquired by the mutated epithelial cells. Our results show, that low expression of E-cadherin in tumours is related to a shorter survival time of patients with CRC.

Another crucial stage of metastasis is tumour cells acquiring the ability to evade the immune system. IL-10, TGF-β, PD-1, and its ligand PD-L1 are potent immunosuppressive molecules produced by innate and adaptive immune cells including T cells, M2-like macrophages, dendritic cells, and natural Killers (NK) cells, as well as tumours including CRC cells [13,46,47]. Previous studies [10,48,49] suggest that blocking PD-1/PD-L1 as therapy does not seem to be effective, since IL-10 is a major compensatory suppression mechanism. Thus, combined blocking of PD-1/PD-L1/TGF-β and disruption of IL-10/IL-10R signalling could enhance survival and reduced the tumour burdon [48,50]. Consistent with our findings, IL-10R signalling is involved in overstimulation of STAT3, enabling tumour cells to grow uncontrollably and develop resistance to apoptosis [11,18,51]. Thus, IL-10 promotes STAT3 activation, and persistent activation is significantly associated with metastasis and a poor prognosis for the patient [18,52]. Furthermore, the IL-10/IL-10R pathway is also connected to the secretion of TGF-β and PD-L1 by M2-TAMs via NF-κB in the TME [53]. An overstimulation of the STAT3/NF-κB pathway by CD163+ M2-TAMs is certainly related to the poor prognosis in human colorectal tumours, as shown by our results [51,54]. In this study, a complex communication network established between immune and malignant cells orchestrated by TAMs has evidently been disrupted by NP3. This was also due to the combination of our NPs with the anti-PD-L1 inhibitor, since we observed a reduction in the expression of IL-10, PD-L1, and TGF-β via STAT3/NF-κB crosstalk in the TME.

When we analysed the expression of PD-L1, CD163, and CXCL12 in human CRC, we observed that a higher expression was related to a poor prognosis for the patient. Our results in mice show that treatment with NP3, alone or combined with anti-PD-L1, decreased the expression of CXCL12, CXCR4, CCL22, PDL-1, CD163, and CD25 in the primary TME. The CXCR4/CXCL12 pathway plays a crucial role in regulating primary tumour growth and metastasis via M2-TAM [55], while CCL22 is associated with infiltration by CD25+ Treg cells for many tumours. In addition, Tregs suppress cell-contact-independent mechanisms, such as secretion of inhibitory cytokines including PDL-1, TGF-β, and IL-10 [56].

Importantly, we found a strong immunomodulatory effect of the anti-PD-L1 + NP3 combination due to the increase of CD8 expression in the TME. Thus, one of the advantages of using the anti-PD-L1 checkpoint blocker in combination with the NP3 is that this treatment has a higher antitumor effect specially for blocking immunosuppression in TME. This therapeutic combination was more effective than free OXA and NP loaded OXA (NP1). In our previous study, we were able to show that NP1 had optimal anti-tumour activity by reducing the drug resistance and increasing the apoptosis in the TME of CRC [57]. Together, these results show that RA-loaded, CHO-coated NPs3 alone or in combination with an anti-PD-L1 immune checkpoint blocker have a strong immunomodulatory effect by upregulating anti-tumour immune cells and, consequently, reducing EMT. This was achieved by downregulation of the STAT3/NF-κB signalling pathways mediated through a loop of immunosuppressive cytokines, such as IL-10/PD-L1/TGF-β via M2-TAM in the TME.

In conclusion, we propose a novel management strategy for aggressive CRC by targeting the EMT via M2-TAMs using RA-loaded, CHO-coated PLGA nanoparticles combined with anti-PD-L1, which, according to our results, showed a potent immunomodulatory effect.

## Funding statement

We acknowledge support by postdoctoral fellowship from Raimundo Fernandes de Araujo Junior by CAPES 88881.119850/2016–01, CNPq 401007/2022-7 and received funding from the EU Program H2020-WIDESPREAD-2017-Twinning ACORN (grant number 807281). We acknowledge support by doctoral fellowship from GAL by CNPq. LJC received funding from the H2020-MSCA-2017-RISE CANCER (grant number 777682). AC received funding from the EU Programs H2020-MSCA-ITN-2019- PRISAR2 [grant number 872860]. CKC received funding from the EU Program H2020-WIDESPREAD-2018-Twinning SIMICA (grant number 852985). TS received funding from EU Program H2020-MSCA-RISE-2019 CONCRETE (grant number 872391). CJC received funding from EU Horizon 2020, the Marie Sklodowska Curie- CARTHAGO (grant number 955335)

## Author contributions

RFAJ designed the experiments. RFAJ, RC, TS, RFG, OV, NFV, and CJC performed the experiments. RFAJ, GAL, RCMP and RC analysed the data. RFAJ, AC, CKC and LC participated in the coordination of study. RFAJ drafted the manuscript. All authors have approved the final version of the manuscript and are accountable for all aspects of this study

## Institutional review board statement

The study was conducted according to the guidelines of the Declaration of Helsinki, and approved by the Institutional Review Board (or Ethics Committee) of the Federal University of Rio Grande do Norte (UFRN), Brasil. Mouse experiments were approved by the Committee on the Ethics of Animal Experiments of the UFRN (CEUA, Permit Number: 170.020/2019). The study related to biopsies obtained from 180 patients undergoing surgical resection of CRC was approved by the institutional committee (No. 030/0030/2006).

## Informed consent statement

Not applicable.

## Data availability statement

The data that support the findings of this study are available from the corresponding author upon reasonable request.

## Declaration of Competing Interest

The authors declare that they have no known competing financial interests or personal relationships that could have appeared to influence the work reported in this paper.

## Appendix Supplementary materials

Image, application 1

Image, application 2

## References

[1] Lin C., Zhang J. (2017). Inflammasomes in inflammation-induced cancer. Front. Immunol..

[2] Wong M.C.S. (2020). Differences in incidence and mortality trends of colorectal cancer worldwide based on sex, age, and anatomic location. Clin. Gastroenterol. Hepatol..

[3] Dulai P.S., Sandborn W.J., Gupta S. (2016). Colorectal cancer and dysplasia in inflammatory bowel disease: a review of disease epidemiology. Pathophysiol. Manag. Cancer Prev. Res..

[4] Ponzoni M., Pastorino F., Di Paolo D., Perri P., Brignole C. (2018). Targeting macrophages as a potential therapeutic intervention: impact on inflammatory diseases and cancer. Int. J. Mol. Sci..

[5] Cersosimo F. (2020). Tumor-associated macrophages in osteosarcoma: from mechanisms to therapy. Int. J. Mol. Sci..

[6] Schultze J.L. (2016). Reprogramming of macrophages–new opportunities for therapeutic targeting. Curr. Opin. Pharmacol..

[7] Martinez F.O., Gordon S. (2014). The M1 and M2 paradigm of macrophage activation: time for reassessment. F1000Prime Rep.

[8] Sumitomo R. (2019). PD-L1 expression on tumor-infiltrating immune cells is highly associated with M2 TAM and aggressive malignant potential in patients with resected non-small cell lung cancer. Lung Cancer.

[9] Anfray C., Ummarino A., Andon F.T., Allavena P. (2019). Current strategies to target tumor-associated-macrophages to improve anti-tumor immune responses. Cells.

[10] Lind H. (2020). Dual targeting of TGF-beta and PD-L1 via a bifunctional anti-PD-L1/TGF-betaRII agent: status of preclinical and clinical advances. J. Immunother. Cancer.

[11] de Araujo Junior R.F. (2020). Ceramide and palmitic acid inhibit macrophage-mediated epithelial-mesenchymal transition in colorectal cancer. Mol. Cell. Biochem..

[12] Cai J. (2019). Tumor-associated macrophages derived TGF-betainduced epithelial to mesenchymal transition in colorectal cancer cells through Smad2,3-4/snail signaling pathway. Cancer Res. Treat..

[13] Xie F., Ling L., van Dam H., Zhou F., Zhang L. (2018). TGF-beta signaling in cancer metastasis. Acta Biochim. Biophys. Sin..

[14] Dorrington M.G., Fraser I.D.C. (2019). NF-kappaB signaling in macrophages: dynamics, crosstalk, and signal integration. Front. Immunol..

[15] Kanda N. (2004). STAT3 is constitutively activated and supports cell survival in association with survivin expression in gastric cancer cells. Oncogene.

[16] Scholz A. (2003). Activated signal transducer and activator of transcription 3 (STAT3) supports the malignant phenotype of human pancreatic cancer. Gastroenterology.

[17] Liu Q. (2017). Factors involved in cancer metastasis: a better understanding to "seed and soil" hypothesis. Mol. Cancer.

[18] Cavalcante R.S. (2021). M2 TAM-associated STAT3/NF-kappaB signalling suppression as major target of immunomodulatory therapy with PLGA-based nanocarriers and anti-PD-L1 in breast cancer. Br. J. Pharmacol..

[19] Chen Q., Ross A.C. (2004). Retinoic acid regulates cell cycle progression and cell differentiation in human monocytic THP-1 cells. Exp. Cell. Res..

[20] Hansen L.A. (2000). Retinoids in chemoprevention and differentiation therapy. Carcinogenesis.

[21] Wang S. (2009). Differential expression of phosphorylated extracellular signal-regulated kinase 1/2, phosphorylated p38 mitogen-activated protein kinase and nuclear factor-kappaB p105/p50 in chronic inflammatory skin diseases. J. Dermatol..

[22] Tsagozis P., Augsten M., Pisa P. (2014). All trans-retinoic acid abrogates the pro-tumorigenic phenotype of prostate cancer tumor-associated macrophages. Int. Immunopharmacol..

[23] Zhou Q. (2017). All-trans retinoic acid prevents osteosarcoma metastasis by inhibiting M2 polarization of tumor-associated macrophages. Cancer Immunol. Res..

[24] Goossens P. (2019). Membrane cholesterol efflux drives tumor-associated macrophage reprogramming and tumor progression. Cell Metab..

[25] Pradel L.C. (2009). ATP-binding cassette transporter hallmarks tissue macrophages and modulates cytokine-triggered polarization programs. Eur. J. Immunol..

[26] Mooberry L.K., Sabnis N.A., Panchoo M., Nagarajan B., Lacko A.G. (2016). Targeting the SR-B1 receptor as a gateway for cancer therapy and imaging. Front. Pharmacol..

[27] Harisa G.I., Alanazi F.K. (2014). Low density lipoprotein bionanoparticles: from cholesterol transport to delivery of anti-cancer drugs. Saudi Pharm. J..

[28] Ribeiro I.S (2021). Poly(epsilon-caprolactone) grafted cashew gum nanoparticles as an epirubicin delivery system. Int. J. Biol. Macromol..

[29] Hunter M.M. (2010). In vitro-derived alternatively activated macrophages reduce colonic inflammation in mice. Gastroenterology.

[30] Honda M., Kadohisa M., Yoshii D., Komohara Y., Hibi T. (2021). Directly recruited GATA6 + peritoneal cavity macrophages contribute to the repair of intestinal serosal injury. Nat. Commun..

[31] Araujo R.F. (2015). Prognostic and diagnostic implications of MMP-2, MMP-9, and VEGF-alpha expressions in colorectal cancer. Pathol. Res. Pract..

[32] Benner B. (2019). Generation of monocyte-derived tumor-associated macrophages using tumor-conditioned media provides a novel method to study tumor-associated macrophages in vitro. J. Immunother. Cancer.

[33] Ambade A. (2016). Hepatocellular carcinoma is accelerated by NASH involving M2 macrophage polarization mediated by hif-1alphainduced IL-10. Oncoimmunology.

[34] Larionova I. (2019). Interaction of tumor-associated macrophages and cancer chemotherapy. Oncoimmunology.

[35] Chen Y., Tan W., Wang C. (2018). Tumor-associated macrophage-derived cytokines enhance cancer stem-like characteristics through epithelial-mesenchymal transition. Onco Targets Ther..

[36] Pettersson F., Colston K.W., Dalgleish A.G. (2001). Retinoic acid enhances the cytotoxic effects of gemcitabine and cisplatin in pancreatic adenocarcinoma cells. Pancreas.

[37] Grunt Th W. (1998). Effects of retinoic acid and fenretinide on the c-erbB-2 expression, growth and cisplatin sensitivity of breast cancer cells. Br. J. Cancer.

[38] Manzanares D., Cena V. (2020). Endocytosis: the nanoparticle and submicron nanocompounds gateway into the cell. Pharmaceutics.

[39] Tenchov B.G., MacDonald R.C., Siegel D.P. (2006). Cubic phases in phosphatidylcholine-cholesterol mixtures: cholesterol as membrane "fusogen". Biophys. J..

[40] Pozzi D. (2012). Transfection efficiency boost of cholesterol-containing lipoplexes. Biochim. Biophys. Acta.

[41] Tang T. (2019). Tumor-specific macrophage targeting through recognition of retinoid X receptor beta. J. Control Release.

[42] Chen Q. (2019). Targeted inhibition of STAT3 as a potential treatment strategy for atherosclerosis. Theranostics.

[43] Wang P., Wu P., Siegel M.I., Egan R.W., Billah M.M. (1995). Interleukin (IL)-10 inhibits nuclear factor kappa B (NF kappa B) activation in human monocytes. IL-10 and IL-4 suppress cytokine synthesis by different mechanisms. J. Biol. Chem..

[44] He Y., de Araujo Junior R.F., Cruz L.J., Eich C. (2021). Functionalized nanoparticles targeting tumor-associated macrophages as cancer therapy. Pharmaceutics.

[45] Kitamura T., Qian B.Z., Pollard J.W. (2015). Immune cell promotion of metastasis. Nat. Rev. Immunol..

[46] Ouyang W., Rutz S., Crellin N.K., Valdez P.A., Hymowitz S.G. (2011). Regulation and functions of the IL-10 family of cytokines in inflammation and disease. Annu. Rev. Immunol..

[47] Zhang Y. (2015). Prognostic significance of programmed cell death 1 (PD-1) or PD-1 ligand 1 (PD-L1) Expression in epithelial-originated cancer: a meta-analysis. Medicine.

[48] Rahimi Kalateh Shah Mohammad G., Ghahremanloo A., Soltani A., Fathi E., Hashemy S.I (2020). Cytokines as potential combination agents with PD-1/PD-L1 blockade for cancer treatment. J. Cell. Physiol..

[49] Lamichhane P. (2017). IL10 Release upon PD-1 blockade sustains immunosuppression in ovarian cancer. Cancer Res..

[50] Jarnicki A.G., Lysaght J., Todryk S., Mills K.H. (2006). Suppression of antitumor immunity by IL-10 and TGF-beta-producing T cells infiltrating the growing tumor: influence of tumor environment on the induction of CD4+ and CD8+ regulatory T cells. J. Immunol..

[51] Grivennikov S.I., Karin M. (2010). Dangerous liaisons: STAT3 and NF-kappaB collaboration and crosstalk in cancer. Cytokine Growth Factor Rev..

[52] Banerjee K., Resat H. (2016). Constitutive activation of STAT3 in breast cancer cells: a review. Int. J. Cancer.

[53] Jiang X. (2019). Role of the tumor microenvironment in PD-L1/PD-1-mediated tumor immune escape. Mol. Cancer.

[54] Kusaba T. (2006). Activation of STAT3 is a marker of poor prognosis in human colorectal cancer. Oncol. Rep..

[55] Domanska U.M. (2013). A review on CXCR4/CXCL12 axis in oncology: no place to hide. Eur. J. Cancer.

[56] Sakaguchi S. (2000). Regulatory T cells: key controllers of immunologic self-tolerance. Cell.

[57] Oliveira A.LC..S.L. (2020). Effect of oxaliplatin-loaded poly (d,l-Lactide-co-Glycolic Acid) (PLGA) nanoparticles combined with retinoic acid and cholesterol on apoptosis, drug resistance, and metastasis factors of colorectal cancer. Pharmaceutics.

