# Supplementary material for: Retinoic acid-loaded PLGA nanocarriers targeting cell cholesterol potentialize the antitumour effect of PD-L1 antibody by preventing epithelial-mesenchymal transition mediated by M2-TAM in colorectal cancer
Source: Transl Oncol. 2023 Feb 27;31:101647. doi: 10.1016/j.tranon.2023.101647 (PMC9989692; doi:10.1016/j.tranon.2023.101647)
Supplement: Supplementary file 1 [file mmc1.docx]

**SUPPLEMENTARY DATA**

**Retinoic Acid-loaded PLGA Nanocarriers targeting cell cholesterol potentialize the antitumour effect of PD-L1 Antibody by Preventing Epithelial-Mesenchymal Transition Mediated by M2-TAM in colorectal cancer.**

Raimundo Fernandes de Araújo Júnior^1,2,3,4,5*^, George A Lira^1,3,5,7^, Timo Schomann^4,5^ ,Rômulo S Cavalcante^1,3^, Natalia Feitosa Vilar^1^, Regina Célia Monteiro de Paula^8^, Raelle Ferreira Gomes^8^, Chih Kit Chung^4,5,9^, Carla Jorquera-Cordero^4,6^, Olena Vepris^5^, Alan B Chan^4^, Luis J. Cruz^5^

1 Cancer and Inflammation Research Laboratory, Department of Morphology, Federal University of Rio Grande do Norte Natal 59072-970, RN, Brazil.

2 Post-Graduation Programme in Structural and Functional Biology, Federal University of Rio Grande do Norte, Natal 59072-970, RN, Brazil;

3 Post-Graduation Programme in Health Science, Federal University of Rio Grande do Norte, Natal 59072-970, RN, Brazil;

4 Percuros B.V., 2333 CL Leiden, The Netherlands;

5 Translational Nanobiomaterials and Imaging, Department of Radiology, Leiden University Medical Center, 2333 ZA Leiden, The Netherlands.

6 Department of Orthopedics, University Medical Center Utrecht, the Netherlands, Heidelberglaan 100, 3584 CX Utrecht

7 League Against Cancer from Rio Grande do Norte, Advanced Oncology Center, 59075-740 Natal, Brazil.

8 Post-Graduation Programme in Chemistry, Federal University of Ceará, Fortaleza, 60440-900, CE, Brazil;

9 JeNaCell GmbH, Winzerlaer Straße 2, 07745 Jena, Germany

* Correspondence: fernandes.araujo@ufrn.br; Tel.: +31655620247

Corresponding author: Raimundo Fernandes de Araújo Júnior, Radiology Department/ Leiden University Medical Centre, Leiden, The Netherlands. Post code: 2333 ZA. +31655620247. araujojr@cb.ufrn.br

**SUPPLEMENTARY FIGURE LEGENDS**


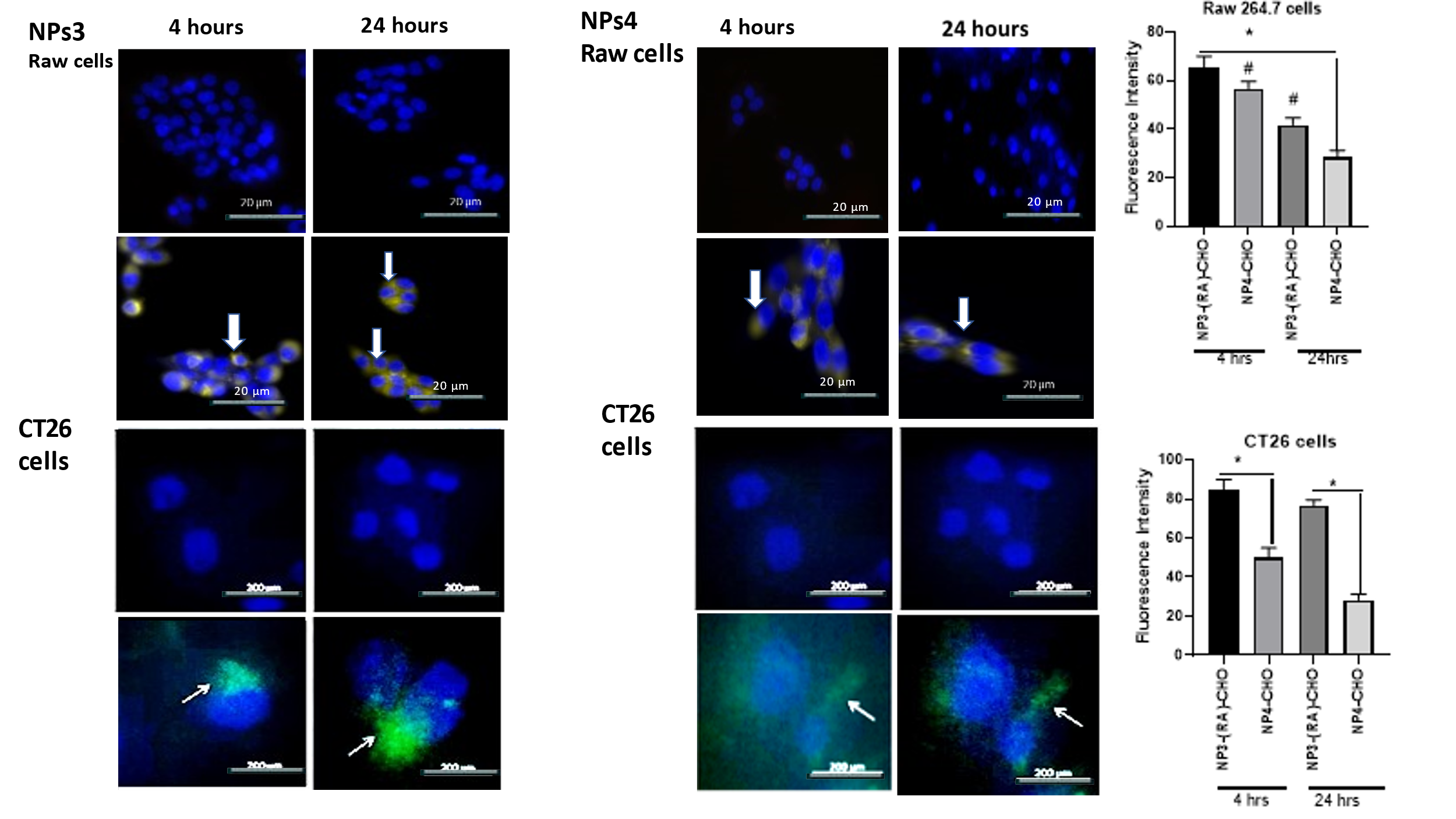


**Supplementary Figure 1s. Internalization assays of nanoparticles by macrophages (RAW 264.7 cells) and CT-26 cells.** Fluorescence images indicate the uptake of NPs3 and NPs4 (green) by RAW 264.7 cells and CT-26 cells after 4 hours and 24 hours. NP8 was excluded from this experiment since they did not contain RA.


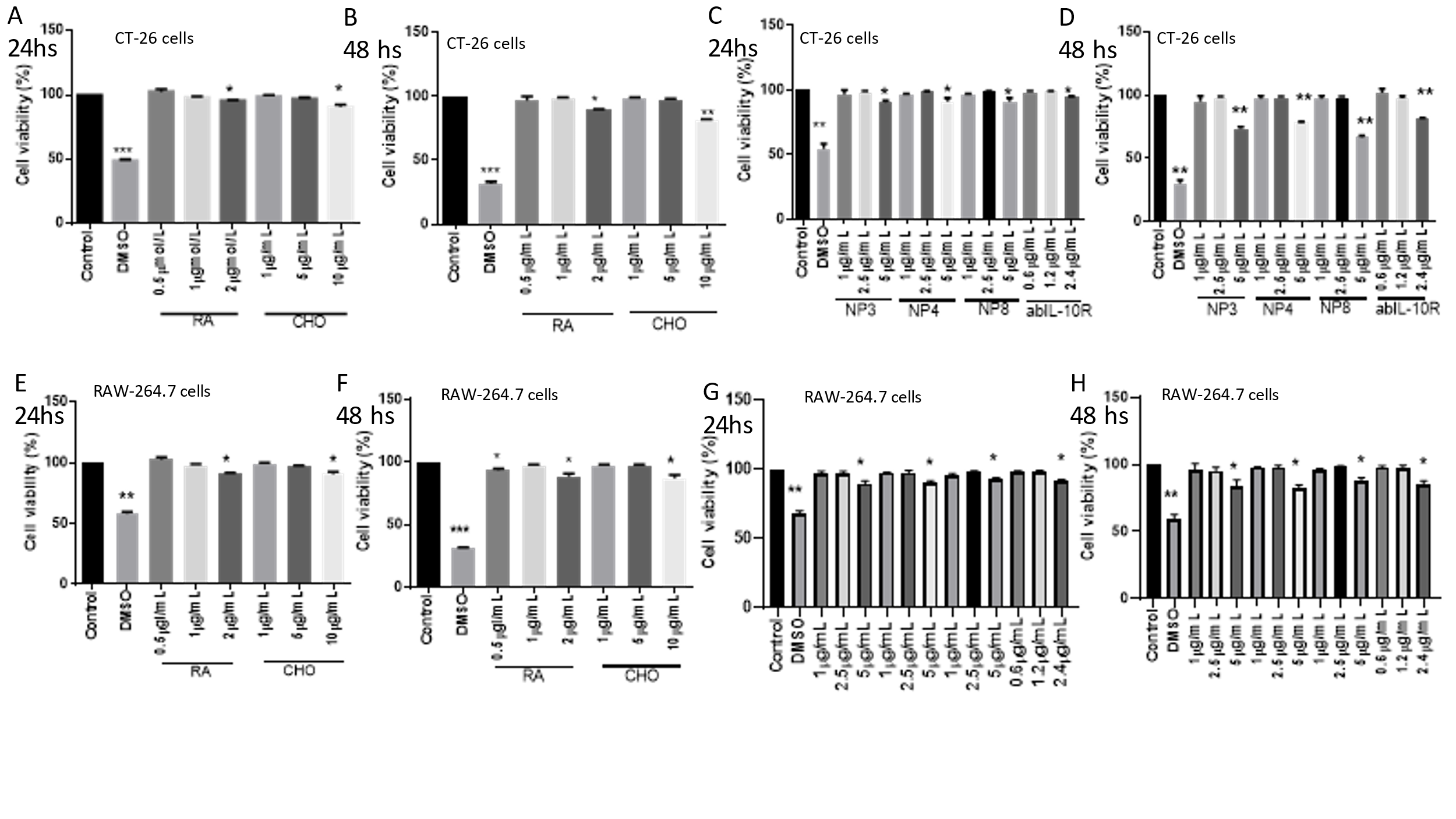


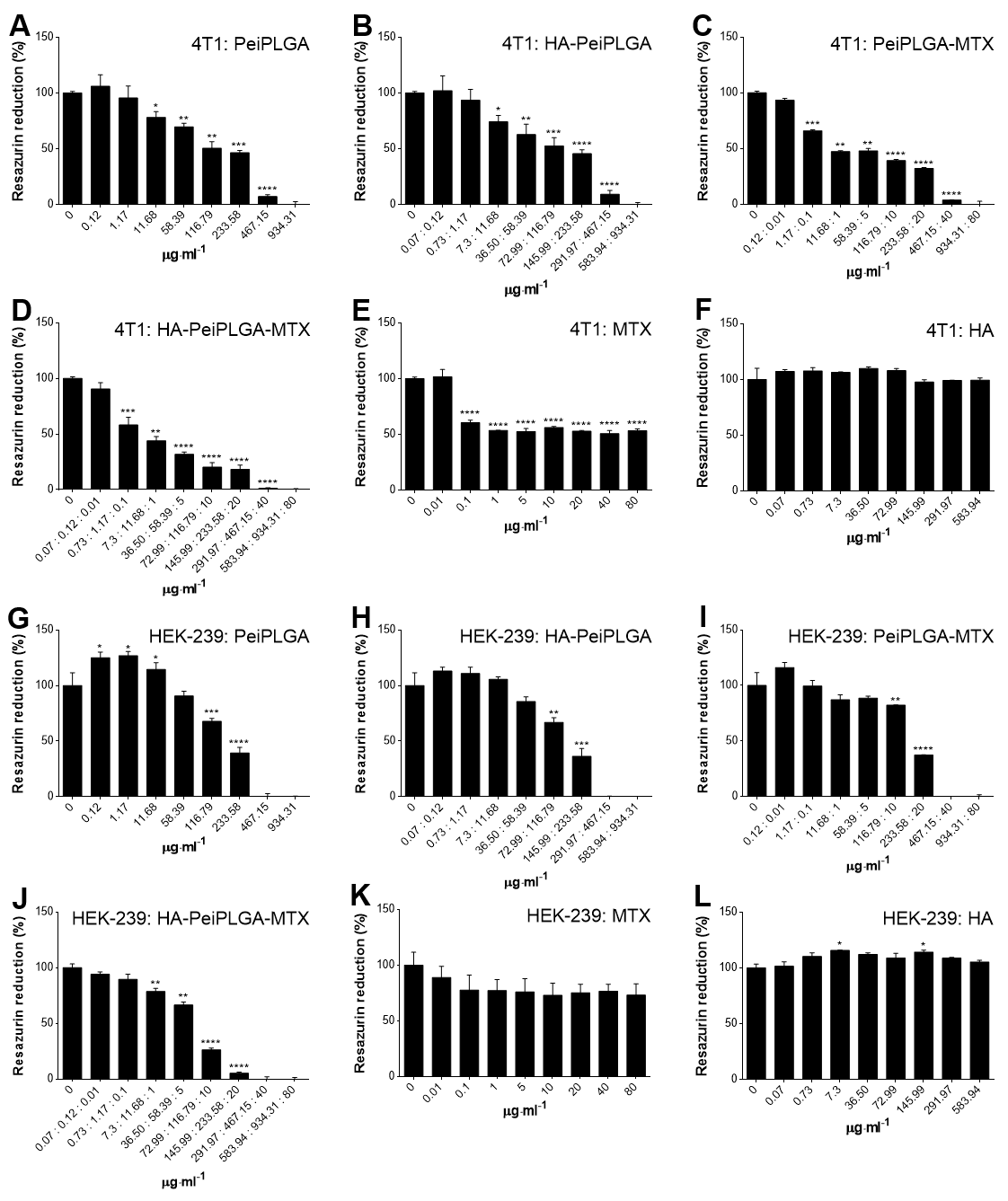
**Supplementary Fig. S2: Cell viability of CT-26 and RAW 264.7 cells after treatment with RA, CHO, NPs 3 (RA)-CHO, NPs 4 -CHO and anti-IL-10R inhibitor for 24 hrs and 48 hrs, assessed by MTS assay.** Cell viability of CT-26 cells treated with increasing concentrations (0.5μg/mL - 2 μg/mL RA) and (1μg/mL - 10μg/mL CHO) after 24 hrs (A) and 48 hrs (B), respectively. Cell viability of CT-26 cells treated with increasing concentrations (1μg/mL - 5 μg/mL NPs3), (1μg/mL - 5 μg/mL NPs4), (1μg/mL - 5 μg/mL NPs8) and (0.6μg/mL – 2.4μg/mL abIL-10R) after 24 hrs (C) and 48 hrs (D), respectively. Cell viability of RAW 264.7 cells treated with increasing concentrations (0.5μg/mL - 2 μg/mL RA) and (1μg/mL - 10μg/mL CHO) after 24 hrs (E) and 48 hrs (F), respectively. Cell viability of RAW 264.7 cells treated with increasing concentrations (1μg/mL - 5 μg/mL NPs3), (1μg/mL - 5 μg/mL NPs4), (1μg/mL - 5 μg/mL NPs8) and (0.6μg/mL – 2.4μg/mL abIL-10R) after 24 hrs (G) and 48 hrs (H), respectively. The data represent the mean ± SEM of 3-6 independent experiments. All p-values were compared to control cells by analysis of variance and the Bonferroni's test, *p<0.05; **p<0.01 ***p<0.001 versus control.


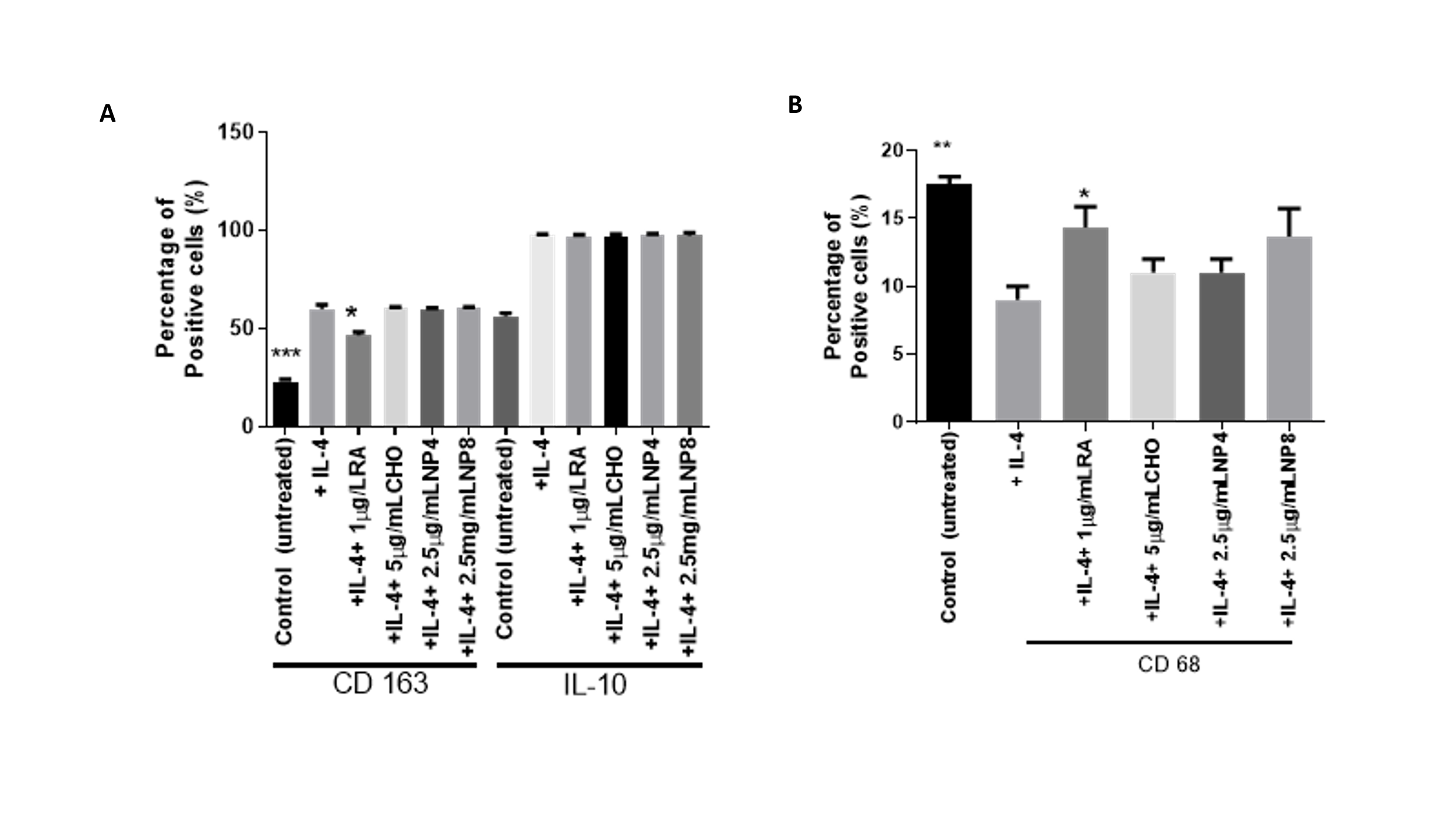


**Supplementary Fig. S3: Modulation of M2-polarized RAW cells by PLGA NPs.** After treatment with 1 μgmol/L RA, 5 μg/mL CHO, 2.5 μg/mL NPs4- CHO and 2.5 μg/mL NPs8- (RA) during the 48 hs polarization cell culture, the IL-4-induced M2-polarized RAW cells were evaluated for labelling for CD163 (A), IL-10 (A) and CD68 (B) by flow cytometry. One-way ANOVA with post-hoc Bonferroni correction; *p<0.05, **p<0.01, ***p<0.001All data are presented as mean ± SD of five independent assays with at least three replicates.
