# Supplementary material for: Retinoic acid-loaded PLGA nanocarriers targeting cell cholesterol potentialize the antitumour effect of PD-L1 antibody by preventing epithelial-mesenchymal transition mediated by M2-TAM in colorectal cancer
Source: Transl Oncol. 2023 Feb 27;31:101647. doi: 10.1016/j.tranon.2023.101647 (PMC9989692; doi:10.1016/j.tranon.2023.101647)
Supplement: Supplementary file 2 [file mmc2.docx]

**SUPPLEMENTARY DATA**

**Retinoic Acid-loaded PLGA Nanocarriers targeting cell cholesterol potentialize the antitumour effect of PD-L1 Antibody by Preventing Epithelial-Mesenchymal Transition Mediated by M2-TAM in colorectal cancer**

Raimundo Fernandes de Araújo Júnior^1,2,3,4,5^*, George A Lira^1,3,5,7^ ,Timo Schomann^4,5^, Rômulo S Cavalcante^1,3^, Natalia Feitosa Vilar^1^, Regina Célia Monteiro de Paula^8^, Raelle Ferreira Gomes^8^, Chih Kit Chung^4,5,9^, Carla Jorquera-Cordero^4,6^, Olena Vepris^5^, Alan B Chan^4^, Luis J. Cruz^5^

^1^  Cancer and Inflammation Research Laboratory, Department of Morphology, Federal University of Rio Grande do Norte Natal 59072-970, RN, Brazil.

^2^  Post-Graduation Programme in Structural and Functional Biology, Federal University of Rio Grande do Norte, Natal 59072-970, RN, Brazil;

^3^ Post-Graduation Programme in Health Science, Federal University of Rio Grande do Norte, Natal 59072-970, RN, Brazil;

^4^ Percuros B.V., 2333 CL Leiden, The Netherlands;

^5^ Translational Nanobiomaterials and Imaging, Department of Radiology, Leiden University Medical Center, 2333 ZA Leiden, The Netherlands.

^6^ Department of Orthopedics, University Medical Center Utrecht, the Netherlands, Heidelberglaan 100, 3584 CX Utrecht

^7^  League Against Cancer from Rio Grande do Norte, Advanced Oncology Center, 59075-740 Natal, Brazil.

^8^ Post-Graduation Programme in Chemistry, Federal University of Ceará, Fortaleza, 60440-900, CE, Brazil;

^9^ JeNaCell GmbH, Winzerlaer Straße 2, 07745 Jena, Germany

* Correspondence: fernandes.araujo@ufrn.br; Tel.: +31655620247.Radiology Department/ Leiden University Medical Centre, Leiden, The Netherlands. Post code: 2333 ZA. +31655620247, fernandes.araujo@ufrn.br

**SUPPLEMENTARY TABLES**

**Supplementary Table 1.** Primer Sequences used for PCR

| **Target** | **5’ → 3’ Forward** | **5’ → 3’ Reverse** | **Amplicon size (pb)** | **Annealing temperature (°C)** |
| --- | --- | --- | --- | --- |
| *β-actin* | AGGCCAACCTGTAAAAGATG | TGTGGTACGAGAGGCATAC | 107 | 50.94 |
| *STAT3* | GGGCCTGGTGTGAACTACTC | GGTATTGCTGCAGGTCGTTG | 564 | 54.79 |
| *NFKB* | CCGTCTGTCTGCTCTCTCT | CGTAGGGATCATCGTCTGCC | 873 | 54.08 |
| *CD-68* | CGCCTAGTCCAAGGTCCAAG | GAAGTGTCCCTTGTCAGGCA | 488 | 55.00 |
| *CD-163* | GGATCTCCGGGATGCTTCTG | CGCCTGCCAGACGAATATCT | 878 | 54.97 |
| *CCL22* | GAGACAACAGTGGTCCCAGG | CTGGCACTGTCAATCCCTGT | 185 | 54.96 |
| *CD8* | GCTCAGTCATCAGCAACTCG | ATCACAGGCGAAGTCCAATC | 197 | 53.48 |
| *CXCR4* | CATGGAACCGATCAGTGTGAG | TGAAGGCCAGGATGAGAACG | 395 | 54.24 |
| CD25 | GGCTATCCCAGTGTGCTCTC | GACTGTTTTTATGGAAAGGT | 534 | 59.22 |
| Cadherin-E | TGATGATGCCCCCAACACTC | CCAAGCCCTTGGCTGTTTTC | 128 | 60.00 |
| Vimentin | TCCAGAGAGAGGAAGCCGAA | CTTTCATACTGCTGGCGCAC | 253 | 59.90 |
| PDL1 | CCAGCCACTTCTGAGCATGA | CAGACAGCAAGAGCCTGTCA | 931 | 60.00 |

**Supplementary** **Table 2.** **Clinicopathological characteristics of CRC patients in relation to Cadherin-E, STAT3 and NFκB expression.**

|  |  | **E-Cadherin** | | | **STAT3** | | | **NFκB** | | |
| --- | --- | --- | --- | --- | --- | --- | --- | --- | --- | --- |
|  |  | Weak | Strong | p | Weak | Strong | p | Weak | Strong | p |
| **Number of patients** |  | 144  (80%) | 36  (20%) |  | 44  (24.4%) | 136  (75.5%) |  | 66  (36.3%) | 114  (63.3%) |  |
|  |  |  |  |  |  |  |  |  |  |  |
| **Lymph nodes status** | **Positive** | 114  (63.4%) | 9  (5%) |  | 4  (2.2%) | 102  (56.6%) | 0.0001 | 19  (10.5%) | 90  (50%) |  |
|  |  |  |  |  |  |  |  |  |  |  |
|  | **Negative** | 30  (16.6%) | 27  (15%) | 0.0001 | 40  (22.2%) | 34  (18.8%) |  | 24  (13.3%) | 47  (26.1%) | 0.0001 |
|  |  |  |  |  |  |  |  |  |  |  |
| **Modified Duke's criteria grade** | **Low (I/II)** | 30  (16.6%) | 3  (1.6%) | 0.005 | 32  (17.7%) | 42  (23.3%) | 0.0001 | 16  (8.8%) | 14  (7.7%) | 0.22 |
|  | **High (III/IV)** | 114  (63.3%) | 33  (18.5%) |  | 12  (6.6%) | 138  (76.6%) |  | 52  (28.8%) | 98  (54.4%) |  |
|  |  |  |  |  |  |  |  |  |  |  |
| **Evolution** | **Alive (free of in treatment)** | 86  (47.7%) | 34  (18.8%) |  | 30  (16.6%) | 26  (14.4%) | 0.0001 | 13  (7.2%) | 53  (29.4%) |  |
|  | **Death (%)** | 58  (32.4%) | 2  (1.1%) | 0.0001 | 14  (7.7%) | 110  (61.1%) |  | 47  (26.1%) | 67  (37.2%) | 0.0001 |

Low expression of Cadherin-E was associated with higher tumor grade and lymph node metastasis while high expression of STAT3 and NFκB was associated with higher tumor grade and lymph node metastasis. Fisher’s Exact = 1, Chi-square = 2.

**Supplementary Table 3.** **Clinicopathological characteristics of CRC patients in relation to CD163, PDL1 and CXCL12 expression.**

|  |  | **CD163** | | | **PDL-1** | | | **CXCL12** | | | |
| --- | --- | --- | --- | --- | --- | --- | --- | --- | --- | --- | --- |
|  |  | Weak | Strong | p | Weak | Strong | p | Weak | Strong | p |  |
| **Number of patients** |  | 74  (41.1%) | 106  (58.8%) |  | 35  (19.4%) | 145  (80.5%) |  | 22  (12.2%) | 158  (87.7%) |  |  |
|  |  |  |  |  |  |  |  |  |  |  |  |
| **Lymph nodes status** | **Positive** | 9  (5%) | 98  (54.4%) | 0.0001 | 16  (8.8%) | 95  (52.7%) |  | 18  (10%) | 145  (80.5%) | 0.05 |  |
|  | **Negative** | 65  (36.1%) | 8  (4.4%) |  | 19  (10.5%) | 50  (27.7%) | 0.03 | 4  (2.2%) | 13  (7.2%) |  |  |
|  |  |  |  |  |  |  |  |  |  |  |  |
| **Modified Duke's criteria grade** | **Low (I/II)** | 58  (32.2%) | 15  (8.3%) | 0.0001 | 28  (15.5%) | 43  (23.8%) |  | 13  (7.2%) | 48  (26.6%) | 0.01 |  |
|  |  |  |  |  |  |  |  |  |  |  |  |
|  | **High (III/IV)** | 16  (8.8%) | 91  (50.5%) |  | 7  (3.8%) | 102  (56.6%) | 0.0001 | 9  (5%) | 110  (61.1%) |  |  |
|  |  |  |  |  |  |  |  |  |  |  |  |
| **Evolution** | **Alive (free of in treatment)** | 55  (30.5%) | 6  (3.3%) | 0.0001 | 32  (17.7%) | 14  (7.7%) |  | 18  (10%) | 53  (29.4%) |  |  |
|  |  |  |  |  |  |  |  |  |  |  |  |
|  | **Death (%)** | 19  (10.5%) | 100  (55.5%) |  | 3  (1.6%) | 131  (72.7%) | 0.0001 | 4  (2.2%) | 105  (58.3%) | 0.0001 |  |
|  |  |  |  |  |  |  |  |  |  |  |  |

High expression of CD163, PDL1 and CXCL12 was associated with higher tumor grade and lymph node metastasis. Fisher’s Exact = 1, Chi-square = 2.
